# Supplementary material for: The effect of behavioral activation play therapy in adolescents with depression: A study protocol for a randomized controlled trial
Source: PLoS One. 2024 Jun 20;19(6):e0304084. doi: 10.1371/journal.pone.0304084 (PMC11189190; doi:10.1371/journal.pone.0304084)
Supplement: S1 File — The original protocol approved by The Regional Committee for Medical and Health Research Ethics. (DOCX) [file pone.0304084.s001.docx]

**研究项目：**

## 行为激活游戏疗法对青少年抑郁症的影响：一个随机对照试验的研究方案

研究负责人：黄小龙

儿少科

广州医科大学附属脑科医院

中国广州510100

电话：020-81268002

电子邮件： XiaoLongHuang0713@hotmail.com

2022-3-17

**行为激活游戏疗法对青少年抑郁症的影响：一个随机对照试验的研究方案**

研究小组

该项目是广州医科大学和广州医科大学附属脑科医院儿童和青少年精神病学系之间的合作。

研究负责人

黄小龙，中国广州医科大学附属脑科医院儿童与青少年精神病学系，广州510100。电子邮件： XiaoLongHuang0713@hotmail.com，电话： 020-81268002

**研究小组成员**

黄小龙，广州医科大学附属脑科医院，中国，广州

周燕玲，广州医科大学附属脑科医院，中国，广州

杨婵娟，广州医科大学附属脑医院，中国，广州

罗薇，广州医科大学附属脑科医院，中国，广州

王冬冬，广州医科大学附属脑科医院，中国，广州

陈宇琦，南方医科大学，中国，广州

罗家成，广州医科大学，中国，广州

**摘要**

本研究中，我们整合了行为激活（Behavioral activation, BA）和游戏化身体活动（Gamified physical activity, GPA），创建了行为激活游戏疗法（Behavioral activation play therapy, BAPT）。这是第一个探索BAPT对青少年抑郁症患者的有效性和适应性的随机对照试验。这项研究将提供有助于减少青少年抑郁症状的证据，并证明治疗在增加身体活动水平、降低非自杀性自残行为的发生率和改善睡眠质量方面的有效性。我们也希望评估BAPT的其他可能的临床效果。

本研究的纳入标准被DSM-5诊断为抑郁症，年龄在12到17岁的患者，排除标准为：(1)参与者被DSM-5诊断有其他精神障碍，包括成瘾障碍、发育障碍、双相情感障碍、物质相关障碍，和精神分裂症（焦虑症除外）；(2)有严重的破坏性或攻击性行为或积极自杀意念的参与者（MADRS＞4的自杀项目得分）；(3)有严重精神病症状（存在疼痛或常见幻觉和/或妄想）的参与者；(4)有临床意义的肺部、内分泌、免疫、心血管疾病（基于辅助检查、体格检查、病史）；(5)参与者不能配合认知功能测试或不适合本研究。

在参与本研究之前，所有受试者及其法定监护人必须充分了解该项目，并签署书面知情同意书。根据预先确定的随机化方案，参与者将被分为干预组和对照组。干预组和对照组分别接受4周BAPT治疗和4周BA治疗。两组的参与者都将按照相同的标准接受治疗和护理，但不会参与任何其他的心理治疗项目。

治疗效果评估将在基线、第2周（基本干预结束时）、第4周（所有高阶干预结束时）、MADRS、BDI-II-C、第8周和第16周分别使用MADRS、BDI-II-C、BADS-SF、ANSAQ、ISI、IPAQ-SF和Likert量表进行。主要结果指标是MADRS评分显示青少年的抑郁症状减轻。

# 研究背景

## 流行病学

2012年至2022年，青少年抑郁症的发病率显著增加。2020年，中国分别有17.2%和7.4%的青少年患有轻度和重度抑郁症状，其终生患病率预计为11%-20%[2]。青少年抑郁症的高患病率是一个公共卫生问题，因为它可以中断发展过程，并对其整体人生阶段 [3,4]产生负面影响。青少年重度抑郁症（Adolescent Major Depressive Disorder, MDD）可产生严重的后果，如辍学、吸毒、自残和自杀，是青少年疾病和残疾的主要原因。目前，青少年抑郁症的临床治疗指南推荐使用心理治疗和药物治疗[5]。然而，心理治疗有许多缺点，如它们往往难以获得，成本昂贵，而且效应量低。另外，一些治疗青少年MDD的药物，如氟西汀和文拉法辛，有较大的副作用，有研究证实它们与增加青少年的自杀意念和行为的风险有关。因此，部分青少年抑郁症患者并不能从药物和心理治疗中获益。因此，有效的干预措施是世界各地心理健康研究人员的一个重要重点。

## 体育活动

近年来，运动疗法因其简单有效而引起全世界的关注。中高强度运动的效果已被证明与抗抑郁治疗[10]相当。因此，临床医生对使用运动治疗青少年MDD[11]采取了越来越积极的态度。2020年，世界卫生组织（WHO）建议儿童和青少年每天进行至少一小时的中高强度的体育活动（Physical Activity, PA），以改善健康和减少抑郁症状[12]。然而，研究表明，只有20%的13-15岁的儿童和青少年遵守了这一建议，[13]患有抑郁症的青少年更不可能达标，因为抑郁症状降低了运动[14]的动机。因此，迫切需要更有效的治疗策略。

.

## 行为激活

2023年，世界卫生组织推荐行为激活（Behavioral activation, BA）作为治疗抑郁症的一种有效的心理治疗形式，并建议不应使用抗抑郁药作为青少年[15]的一线治疗方法。BA在临床上与认知行为疗法一样有效，[16]但成本降低了21%，而且更简单，易于管理。虽然BA是一种治疗青少年抑郁症的有效治疗方法，但它很难在青少年人群中进行，而且其效应量已被发现是[17]不足的。因此，精神卫生工作者已经尝试将BA与其他治疗技术相结合，对青少年抑郁症患者取得更好的治疗结果 [18-20]。例如，BA与数字网络技术的结合受到了青少年[21,22]的广泛欢迎，但这个项目导致了屏幕时间的增加，从而有可能会导致社交和身体活动的减少，伴随久坐行为[23]的增加。此外，有研究证明每天的屏幕时间超过两小时的个体[24]的抑郁风险增加。

## BA与PA结合

BA包括PA可以改善情绪，这对抑郁患者有益，同时增加定期运动[16]的数量。研究表明，BA干预联合PA治疗完成率高，可显著减轻患者的抑郁症状。BA和PA在技术和方法方面具有高度一致的一致性，如自我监控、目标设定和解决问题的技术。结合它们可以克服单独使用每种干预措施的局限性，并降低[25]治疗后抑郁症的复发率.

虽然有研究表明BA联合PA对改善成人抑郁症状是可行和可接受的，但对青少年进行的研究较少。这可能是因为不同年龄组选择了不同形式的PA。与传统的PA形式相比，青少年更倾向于选择游戏形式的PA[26]。因此，在开发用于这类[27]人群的疗法时，增加体育活动的快乐的程度被认为是至关重要的。研究表明，使用游戏化来促进体育活动可以更好地接受[28]，这表明关注游戏化体育活动（GPA）的干预可能是减少青少年[29]久坐行为和抑郁风险的一种实用方法。在大多数青少年中，游戏是PA干预的一个重要因素，而以游戏为中心的活动的整合可能是提高PA水平和改善社会联系的有效方法。事实上，鼓励青少年参与积极、刺激和冒险的游戏可以使他们能够独立地测试自己的能力，从而提高社会弹性[30-32]。利用竞争和奖励可能有助于促进体育活动，以及其他更具创新的方法[33]。

## 研究目标

迄今为止，在中国临床环境中，尚无实证研究证实将 GPA 纳入 BA的干预措施以改善抑郁症青少年抑郁症状的效果。因此，目前还没有关于抗抑郁效果所必需的运动参数的指南。为了解决这个问题，我们的研究小组将BA和GPA整合到行为激活游戏治疗（BAPT）中，并开展一项随机对照试验（RCT）来评估BAPT治疗青少年抑郁症的疗效。我们计划评估BAPT在增加身体活动水平、减少非自杀性自伤行为和改善睡眠质量方面的有效性。我们也希望从临床应用方面评估这种治疗的适应性。

## 假设

1.BAPT（干预组）在缓解抑郁症状（对照组）和提高青少年抑郁症状方面比BA的治疗依从性更有效。

2.BAPT（干预组）在增加身体活动水平、减少非自杀性自伤行为和改善睡眠质量方面比BA（对照组）更有效。

**研究设计**

## 研究设计和设置

本研究为双臂单盲随机对照试验。干预组和对照组将分别接受9次BAPT干预和9次BA干预，因为青少年抑郁症患者住院通常住院时间相对较短，本研究的参与者将会在住院期间完成前4次干预，剩下的5次干预将在其出院后返回病房完成。

**Fig.1**为研究周期。**Fig.2**为本研究的流程图。该研究将由广州医科大学附属脑科医院儿少科和学术伦理委员会监督。

### Fig.1 研究周期


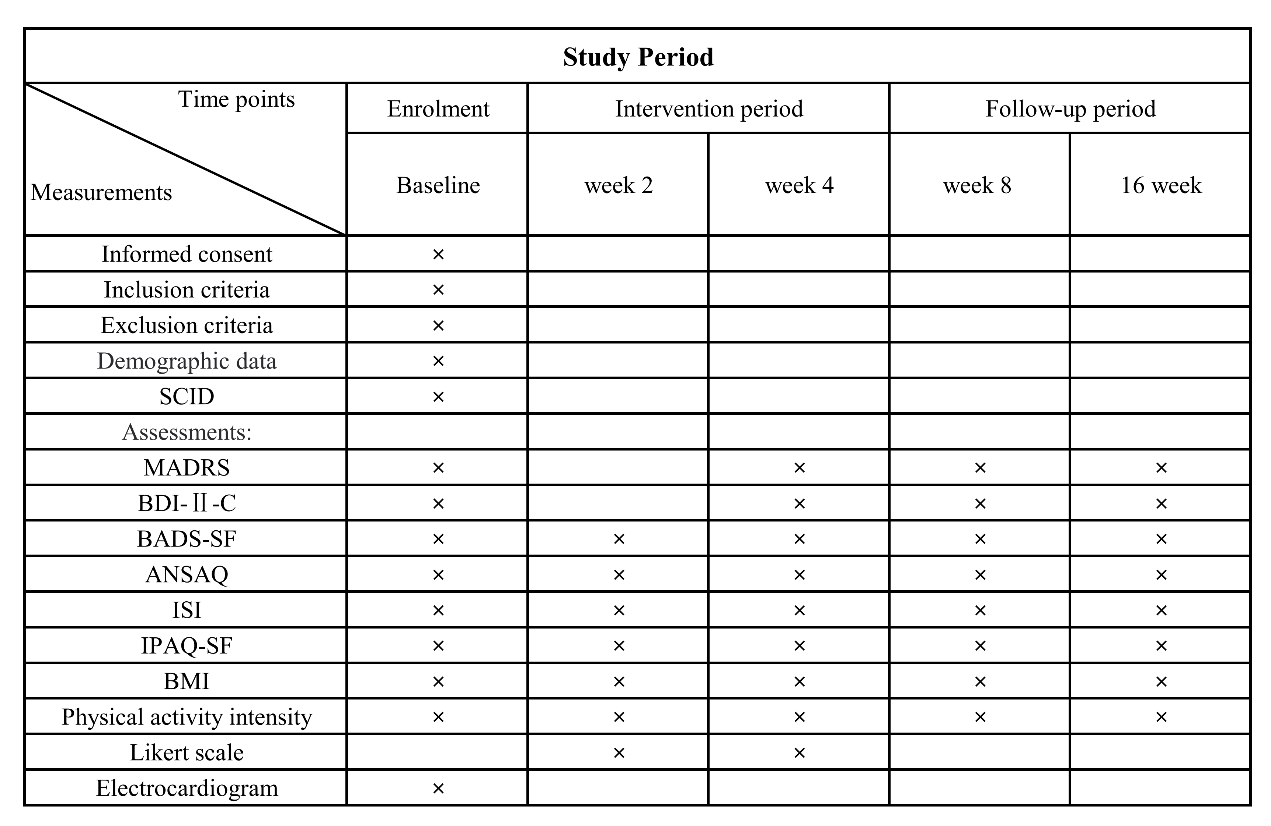


缩写： BMI：体重指数；MADRS：蒙哥马利-阿斯伯格抑郁评定量表；BDI-Ⅱ-C：贝克抑郁量表第二版中文版；BADS-SF：抑郁行为激活量表简表；ANSAQ：青少年非自杀性自杀自伤害评估问卷；ISI：失眠严重程度指数；IPAQ-SF：国际体育活动量表简表；Physical activity intensity：收集有关卡路里消耗（千卡）和代谢当量的数据；Likert scale：评估患者及其家属对干预的接受程度。

### 图2：研究流程图


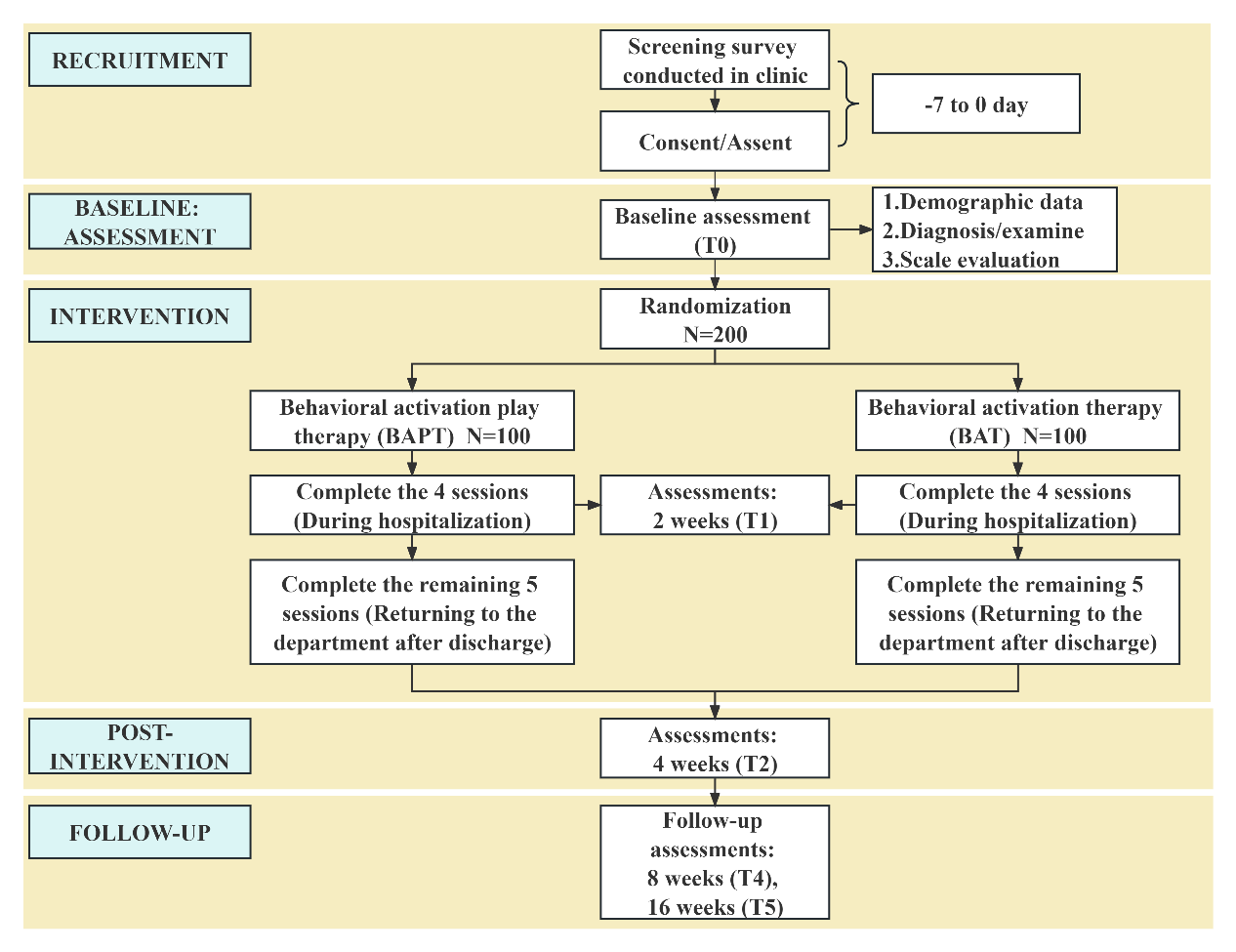


*在研究期间，每周收集有关参与者的身体强度的数据。

**招募**

研究将在广州医科大学附属脑科医院儿少科招募患者。在筛选之前，研究人员将向参与者及其法定监护人解释研究过程，以及研究的重要性、风险和益处。所有参与者及其法定监护人将自愿参与研究，并签署知情同意书，并可在研究过程中随时退出。本研究中提供的干预措施是免费的。

## 受试者选择

受试者是由两名资深精神病学专家根据DSM-5诊断为抑郁症的。Table 1列出了青少年抑郁症的纳入和排除标准。

### Table 1 纳排标准

| **受试者资格** | |
| --- | --- |
| **纳入**  **标准**  **排除**  **标准** | 1. 符合DSM-5诊断无精神病特征的抑郁症的标准； 2. MADRS得分≥12； 3. 12-17岁的住院患者； 4. 智力水平在正常范围内、感知正常、自我表达能力正常、能完成基线量表评估的参与者； 5. 由参与者及其法定监护人签署的知情同意书。 6. 有DSM-5诊断为其他精神障碍的参与者，包括成瘾障碍、发育障碍、双相情感障碍、物质相关障碍和精神分裂症（焦虑症除外）； 7. 有严重破坏性或攻击性行为，或积极自杀意念的参与者（MADRS>4的自杀项目得分）； 8. 有严重精神病症状（存在疼痛或常见幻觉和/或妄想）的参与者； 9. 有临床意义的肺部、内分泌、免疫、心血管疾病的参与者（基于辅助检查、体格检查、病史）； 10. 根据研究小组，参与者不能配合认知功能测试或不适合本研究。 |

缩写：DSM-5：《精神障碍诊断与统计手册》；MADRS：蒙哥马利-阿斯伯格抑郁评定量表。

## 随机化

随机序列将由一名特别招募的工作人员生成，使用SPSS生成一个随机数表。随机的参数和分组将作为机密数据密封在一个不透明的信封中。随机序列和分组数据将由一个指定的人保存，并在选择符合标准的受试者进入研究并签署知情同意书后打开信封。研究者将被告知参与者的治疗分配，参与者将根据预先定义的随机化方案进行随机分配。

**盲法**

本研究将采用单盲方法，因为在此干预中，参与者和卫生保健专业人员不能对研究条件视而不见。因此，为了尽量减少信息偏差的影响，管理结果测量的评估者将不知道组的分配。为了保持研究人员对分组分配的盲目性，同一位研究人员不会对同一指标进行后续测量。

## 干预措施

所有的干预措施都将以小组的形式进行，并由经验丰富的心理治疗师提供。所有参与者将使用电子运动腕带（北京小米科技有限公司，产品编号：小米Band 7pro，M2140B1）来记录他们每天的体育活动强度。电子运动腕带利用光电电容脉冲波追踪（PPG）和加速度传感器来测量青少年[34-37]的身体活动强度。在研究期间，参与者将只接受常规的临床药物治疗和护理，并且将不会参与任何其他的心理治疗项目。

## 干预组：BAPT

干预组将接受9次BAPT治疗，每周两次，每次60分钟。每个课程将包括BA (30分钟）和GPA (30分钟）。BA程序改编自Kellett等人的方案[38]和Lejuez等人[39]，并根据实际情况进行适当的修改。在BA课程中，参与者将收到关于BA核心原则的信息，可概括为以下九个主题：(1)BA的启动：价值观和联系；(2)BA模式和情绪监控；(3)获得激励：目标导向的行为；(4)激活：情境-行动-情绪；(5)解决问题的技能；(6)目标设定和调整；(7)识别障碍和克服回避；(8)思考：担忧、反思；(9)每个课程将包含一个主题，与该主题相关的GPA将在BA课程之后完成。BAPT方案如表2所示。所有的GPA都是由研究团队设计或修改的，具有竞争力、冒险、互动，并遵循符合青少年[26]身心发展特征的某些规则。例如，“猫捉老鼠”是一种令人兴奋的追逐游戏，参与者被要求从三种角色中选择一种：一对参与者是猫和老鼠，而其他的是围栏。所有的围栏都形成了一个圆圈，这样参与者之间的距离大约为1米。鼠标在圆圈内，猫在外面，当游戏开始时，猫试图进入圆圈以抓住鼠标。为了避免被捕获，鼠标可以通过触摸该参与者来选择一个栅栏。然后，原来的栅栏变成了猫，原来的猫变成了老鼠，循环继续，直到猫抓住了老鼠。游戏既有趣又具有挑战性，因为游戏参与者的角色在不断变化。在使用电子运动腕带进行测试后，研究人员将所有的GPA评为中等到高强度的身体活动。因此，我们制定了一个完整的风险预防方案，旨在保护参与者的安全。如果发生了不良事件，它将被记录并报告给医院伦理委员会。

每节课结束后，参与者将接受家庭作业，他们将被鼓励安排活动（社会运动游戏等）。在接下来的一周里，他们感觉愉快，有奖励，并提供一种控制感，并监控这些活动对他们个人情绪的影响。研究人员将每周收集一次家庭作业。

### Table 2 BAPT的方案

| 时间 | 会话 | 课程理论 | 游戏型体育活动 |
| --- | --- | --- | --- |
| 第1 - 2周  （住院期间） | 1 | 启动BA：价值观和连接的感觉 | 大风吹 |
|  | 2 | BA模型和情绪监测 | 气球导弹 |
|  | 3 | 获得激励：目标导向的行为 | 巨人的帽子 |
|  | 4 | 激活：情境-行动-情绪 | 跨越障碍 |
| 第3至4周  （出院后返回科室进行） | 5 | 解决问题的技能 | 脚掌传物 |
|  | 6 | 目标设定和调整 | 蒙眼沟通 |
|  | 7 | 识别障碍并克服回避 | 猫抓老鼠 |
|  | 8 | 思考：担心，反复思考 | 拍膝游戏 |
|  | 9 | 预防复发 | 蚁后游戏 |

缩写：第1-2周：完成第1-4周阶段；第3-4周：完成第5-9周阶段。

## 对照组：BA

对照组的干预与干预组的BA相同，但没有GPA部分。由于延长了讨论时间，每节会议将持续60分钟。家庭作业将与干预组相同，但没有鼓励参加社会运动游戏的建议。

## 干预保真度

BA或BAPT干预措施将由从广州医科大学附属脑科医院的临床工作人员中招募的心理治疗师提供。所有的心理治疗师都将完成一个为期两周的一致性培训计划。在整个研究过程中，将由一名高级临床心理学家进行小组监督，并将记录所有的疗程，以便评估治疗的依从性。

## 安全和监测

在每次干预阶段之前，受试者将完成由临床评分的蒙哥马利-阿斯伯格抑郁评定量表（MADRS）的第10项自杀风险条目。如果得分为>4，受试者将暂时退出研究。在干预过程中，如果受试者表现出严重的自残或自杀行为，他们也将被暂时退出研究。随后，项目团队中的医生将进行自杀危机干预和随访，然后就任何事件组织专家讨论，以避免复发。

## 疗效评价

从基线到第16周，研究人员将利用MADRS评分作为评估抑郁症状变化的主要结局指标。完成10个得分从0到6个[40]的项目，总分为0-60分。抑郁症状按以下评分分为极端、重度、中度、轻度：分别为MADRS > 35、30≤MADRS < 35、22≤MADRS < 30、12≤MADRS < 22、MADRS < 12表示无抑郁症状。与基线相比，MADRS总分降低50%将被定义为显著的抗抑郁反应，而减少20%将被定义为改善。

次要结局指标包括： (1)贝克抑郁量表上的自评抑郁症状，中文第二版(BDI-II-C)：该量表具有较高的内部一致性，反映了过去两周抑郁的严重程度。它包含21个项目，每个项目都是0-3分，总得分为0-63分。抑郁水平根据以下评分范围分别分为29-63、20-28、14-19分，0-13分分为无抑郁[41]。(2)抑郁行为激活量表简表（BADS-SF）：该量表由9个项目组成，测量过去一周内行为激活的变化。激活子量表包括问题1、2、3、4、5和9，而回避子量表包括问题6，7和8。该量表采用了一种7级的评分方法，范围从0（根本没有）到6（完全）。一个项目的得分越高，就表示有一个更接近项目陈述的答案。该量表具有稳健的信度和效度[42](3)青少年非自杀性自残评估问卷（ANSAQ）：该问卷分为行为问卷（12项）和功能问卷（19项）评价自残行为[43]。它使用李克特5分量表，其中“1到5”分别对应于“否，偶尔，有时，经常，总是”。得分越高，自残的程度就越严重。该问卷具有较高的内部一致性，而行为问卷的内部一致性为0.921。在本研究中，我们将只使用问卷的行为维度（共12个问题）来评估抑郁的青少年是否存在自残行为。(4)失眠严重程度指数（ISI）：它被用于评估患者失眠的严重程度。根据对0~4个[44]的7个项目的回答，总分为0-28分。根据评分指南，轻度、中度、重度临床失眠症评分分别为8-14、15-21、22-28分，低于7分为非临床失眠症。(5)国际体育活动问卷-短表格（IPAQ-SF）：这个短表格记录了四个强度水平的活动： 1)高强度的活动，如有氧运动，2)中等强度的活动，如休闲骑自行车，3)步行，4)静坐[45]。这四种强度水平（除了静坐）可以被确定和报告为每周的MET分钟。IPAQ-SF具有良好的内部一致性。(6)定制5分Likert评分接受度问卷：评估参与者及其家属对干预的接受度。

在基线，第2周（基本干预的结束），第4周（所有高阶干预的结束），第8周和16周，两组参与者将评估相应的尺度评估抑郁程度、行为激活水平、非自杀自伤行为和睡眠质量分数。我们还将计算各组各维度在每个时间点的症状改善情况，然后比较两组之间的差异。所有参与者在完成随访后都将获得一个电子运动腕带作为奖励。

## 人口统计学和临床数据

人口统计数据（年龄、性别、职业、文化、居住地、家庭结构、经济收入等）、在筛查阶段将收集医疗费用、生长史、烟草、酒精和其他精神活性物质使用史、精神症状和住院时间。所有参与者及其家属将自愿参与并签署知情同意书。

## 辅助检查

在基线评估时，将完成心电图、血液常规分析和临床治疗的生化指标。

## 体育活动强度

代谢当量（MET）可以用来表示体力活动的相对能量代谢水平。根据练习的强度，可以分为轻、中等和重级，可以分别用0-3、3-6和6分以上表示。

研究人员将每周收集一次关于参与者的身体活动强度的数据。每周完成家庭作业和中等强度锻炼150分钟或以上的参与者将获得小礼物以获得激活和积极强化。电子运动腕带将由项目组购买和分发，以确保监测的一致性。

## 数据管理

病例报告表（CRF）将用于记录所有参与者的人口统计信息和临床症状，随后将通过双重数据输入过程存储在数据库中。研究领导的主要职责将是确保数据输入过程的完整性、准确性和及时性。为了不披露参与者的隐私，研究人员将删除与该研究无关的姓名、电话号码和地址。这些数字将被用来识别参与者。此外，最初的CRF将在整个研究过程中安全存储，并将由项目负责人授权访问。一个专门的医生(教授。周燕玲)将监测数据的完整性、一致性和合理性。

## 统计分析

### 样本量计算

以研究对象的抑郁症状评分作为主要结果指标，双侧α=为0.05，置信区间为90%。在之前的一项关于BA联合传统运动疗法效果的研究中，干预组在基线和16周随访期的抑郁评分分别为27.3 ± 8.0和16.0±9.5[46]。因此，我们预计本研究实验组的抑郁评分较基线降低11.3分，比对照组降低4.8分。我们使用PASS 15软件计算实验组的样本量：N1 = N2 = 84。考虑10%-20%失访，干预组和对照组需要的参与者数量分别约为100人。

## 数据分析

本研究基于意向治疗原则进行，SPSS 28.0将用于数据分析。首先，为了确保随机化后的可比性，我们将在基线时比较两组间的差异。当数据为正态分布时，连续变量采用t检验进行分析，非正态分布的数据采用Mann-Whitney U检验进行分析。各组间在社会人口统计学和临床变量方面的基线差异将使用分类变量的卡方检验和连续数据的方差分析（ANOVA）进行评估。为了比较干预效果，我们将使用混合效应回归模型。组（BAPT或BA）将作为受试者间因素，时间（基线、治疗后和随访）将作为受试者内因素。我们将利用Cohen's d来实现组内和组间的分析。P<为0.05设为差异有统计学意义。

资金支持

本研究由广州科技计划项目（资助号：205171098044）、广州市医学重点学科(Guangzhou Municipal Key Discipline in Medicine (2021-2023))、广州高水平临床重点专科(Guangzhou High-level Clinical Key Specialty)、广州研究型医院(Guangzhou Research-oriented Hospital)联合资助。

**参考文献**

1. Thapar A, Eyre O, Patel V, Brent D. Depression in young people. The Lancet. 2022;400(10352):617-31. doi: <https://doi.org/10.1016/S0140-6736(22)01012-1>.

2. Press BSSA. China National Mental Health Development Report (2019-2020) Blue Book2021.

3. Clayborne ZM, Varin M, Colman I. Systematic Review and Meta-Analysis: Adolescent Depression and Long-Term Psychosocial Outcomes. Journal of the American Academy of Child and Adolescent Psychiatry. 2019;58(1):72-9. Epub 2018/12/24. doi: 10.1016/j.jaac.2018.07.896. PubMed PMID: 30577941.

4. Goldstein BI, Korczak DJ. Links Between Child and Adolescent Psychiatric Disorders and Cardiovascular Risk. The Canadian journal of cardiology. 2020;36(9):1394-405. Epub 2020/07/07. doi: 10.1016/j.cjca.2020.06.023. PubMed PMID: 32628978.

5. Summary of the clinical practice guideline for the treatment of depression across three age cohorts. The American psychologist. 2022;77(6):770-80. Epub 2021/11/30. doi: 10.1037/amp0000904. PubMed PMID: 34843274.

6. Oberste M, Medele M, Javelle F, Lioba Wunram H, Walter D, Bloch W, et al. Physical Activity for the Treatment of Adolescent Depression: A Systematic Review and Meta-Analysis. Frontiers in physiology. 2020;11:185. Epub 2020/04/09. doi: 10.3389/fphys.2020.00185. PubMed PMID: 32265725; PubMed Central PMCID: PMCPMC7096373.

7. Patton GC, Coffey C, Romaniuk H, Mackinnon A, Carlin JB, Degenhardt L, et al. The prognosis of common mental disorders in adolescents: a 14-year prospective cohort study. Lancet (London, England). 2014;383(9926):1404-11. Epub 2014/01/21. doi: 10.1016/s0140-6736(13)62116-9. PubMed PMID: 24439298.

8. Thapar A, Collishaw S, Pine DS, Thapar AK. Depression in adolescence. Lancet (London, England). 2012;379(9820):1056-67. Epub 2012/02/07. doi: 10.1016/s0140-6736(11)60871-4. PubMed PMID: 22305766; PubMed Central PMCID: PMCPMC3488279.

9. Zhang Y, Li G, Liu C, Guan J, Zhang Y, Shi Z. Comparing the efficacy of different types of exercise for the treatment and prevention of depression in youths: a systematic review and network meta-analysis. Frontiers in psychiatry. 2023;14:1199510. Epub 2023/06/19. doi: 10.3389/fpsyt.2023.1199510. PubMed PMID: 37333923; PubMed Central PMCID: PMCPMC10272399.

10. Balchin R, Linde J, Blackhurst D, Rauch HL, Schönbächler G. Sweating away depression? The impact of intensive exercise on depression. Journal of affective disorders. 2016;200:218-21. Epub 2016/05/04. doi: 10.1016/j.jad.2016.04.030. PubMed PMID: 27137088.

11. Radovic S, Melvin GA, Gordon MS. Clinician perspectives and practices regarding the use of exercise in the treatment of adolescent depression. Journal of sports sciences. 2018;36(12):1371-7. Epub 2017/09/26. doi: 10.1080/02640414.2017.1383622. PubMed PMID: 28945524.

12. WHO Guidelines Approved by the Guidelines Review Committee. WHO Guidelines on Physical Activity and Sedentary Behaviour. Geneva: World Health Organization

© World Health Organization 2020.; 2020.

13. Hallal PC, Andersen LB, Bull FC, Guthold R, Haskell W, Ekelund U. Global physical activity levels: surveillance progress, pitfalls, and prospects. Lancet (London, England). 2012;380(9838):247-57. Epub 2012/07/24. doi: 10.1016/s0140-6736(12)60646-1. PubMed PMID: 22818937.

14. Wang DQ, Zhang JJ, Chen JN, Li RY, Luo YX, Deng W. Exergames improves cognitive functions in adolescents with depression: study protocol of a prospective, assessor-blind, randomized controlled trial. BMC psychiatry. 2023;23(1):507. Epub 2023/07/14. doi: 10.1186/s12888-023-04967-7. PubMed PMID: 37442973; PubMed Central PMCID: PMCPMC10339627.

15. WHO. Depressive disorder (depression) 2023. Available from: <https://www.who.int/news-room/fact-sheets/detail/depression>.

16. Pass L, Lejuez CW, Reynolds S. Brief Behavioural Activation (Brief BA) for Adolescent Depression: A Pilot Study. Behavioural and cognitive psychotherapy. 2018;46(2):182-94. Epub 2017/08/02. doi: 10.1017/s1352465817000443. PubMed PMID: 28756787.

17. Cuijpers P, Karyotaki E, Ciharova M, Miguel C, Noma H, Stikkelbroek Y, et al. The effects of psychological treatments of depression in children and adolescents on response, reliable change, and deterioration: a systematic review and meta-analysis. European child & adolescent psychiatry. 2023;32(1):177-92. Epub 2021/10/07. doi: 10.1007/s00787-021-01884-6. PubMed PMID: 34611729; PubMed Central PMCID: PMCPMC9908674.

18. Grudin R, Ahlen J, Mataix-Cols D, Lenhard F, Henje E, Månsson C, et al. Therapist-guided and self-guided internet-delivered behavioural activation for adolescents with depression: a randomised feasibility trial. BMJ open. 2022;12(12):e066357. Epub 2022/12/27. doi: 10.1136/bmjopen-2022-066357. PubMed PMID: 36572500; PubMed Central PMCID: PMCPMC9806095.

19. Davidson TM, Yuen EK, Felton JW, McCauley J, Gros KS, Ruggiero KJ. Feasibility assessment of a brief, web-based behavioral activation intervention for adolescents with depressed mood. International journal of psychiatry in medicine. 2014;48(1):69-82. Epub 2014/10/31. doi: 10.2190/PM.48.1.f. PubMed PMID: 25354927.

20. Van Voorhees BW, Watson N, Bridges JF, Fogel J, Galas J, Kramer C, et al. Development and pilot study of a marketing strategy for primary care/internet-based depression prevention intervention for adolescents (the CATCH-IT intervention). Primary care companion to the Journal of clinical psychiatry. 2010;12(3). Epub 2010/10/15. doi: 10.4088/PCC.09m00791blu. PubMed PMID: 20944776; PubMed Central PMCID: PMCPMC2947535.

21. Bhattacharya A, Nagar R, Jenness J, Munson SA, Kientz JA. Designing Asynchronous Remote Support for Behavioral Activation in Teenagers With Depression: Formative Study. JMIR formative research. 2021;5(7):e20969. Epub 2021/07/14. doi: 10.2196/20969. PubMed PMID: 34255665; PubMed Central PMCID: PMCPMC8317030.

22. Grudin R, Vigerland S, Ahlen J, Widström H, Unger I, Serlachius E, et al. "Therapy without a therapist?" The experiences of adolescents and their parents of online behavioural activation for depression with and without therapist support. European child & adolescent psychiatry. 2023:1-10. Epub 2023/01/18. doi: 10.1007/s00787-023-02142-7. PubMed PMID: 36650254; PubMed Central PMCID: PMCPMC9844942.

23. Nakshine VS, Thute P, Khatib MN, Sarkar B. Increased Screen Time as a Cause of Declining Physical, Psychological Health, and Sleep Patterns: A Literary Review. Cureus. 2022;14(10):e30051. Epub 2022/11/17. doi: 10.7759/cureus.30051. PubMed PMID: 36381869; PubMed Central PMCID: PMCPMC9638701.

24. Liu M, Wu L, Yao S. Dose-response association of screen time-based sedentary behaviour in children and adolescents and depression: a meta-analysis of observational studies. British journal of sports medicine. 2016;50(20):1252-8. Epub 2015/11/11. doi: 10.1136/bjsports-2015-095084. PubMed PMID: 26552416; PubMed Central PMCID: PMCPMC4977203.

25. Farrand P, Pentecost C, Greaves C, Taylor RS, Warren F, Green C, et al. A written self-help intervention for depressed adults comparing behavioural activation combined with physical activity promotion with a self-help intervention based upon behavioural activation alone: study protocol for a parallel group pilot randomised controlled trial (BAcPAc). Trials. 2014;15:196. Epub 2014/06/03. doi: 10.1186/1745-6215-15-196. PubMed PMID: 24886116; PubMed Central PMCID: PMCPMC4061537.

26. Nijhof SL, Vinkers CH, van Geelen SM, Duijff SN, Achterberg EJM, van der Net J, et al. Healthy play, better coping: The importance of play for the development of children in health and disease. Neuroscience and biobehavioral reviews. 2018;95:421-9. Epub 2018/10/03. doi: 10.1016/j.neubiorev.2018.09.024. PubMed PMID: 30273634.

27. Kagawa F, Yokoyama S, Takamura M, Takagaki K, Mitsuyama Y, Shimizu A, et al. Decreased physical activity with subjective pleasure is associated with avoidance behaviors. Scientific reports. 2022;12(1):2832. Epub 2022/02/20. doi: 10.1038/s41598-022-06563-3. PubMed PMID: 35181696; PubMed Central PMCID: PMCPMC8857298.

28. Mazeas A, Duclos M, Pereira B, Chalabaev A. Evaluating the Effectiveness of Gamification on Physical Activity: Systematic Review and Meta-analysis of Randomized Controlled Trials. Journal of medical Internet research. 2022;24(1):e26779. Epub 2022/01/05. doi: 10.2196/26779. PubMed PMID: 34982715; PubMed Central PMCID: PMCPMC8767479.

29. Kandola A, Ashdown-Franks G, Hendrikse J, Sabiston CM, Stubbs B. Physical activity and depression: Towards understanding the antidepressant mechanisms of physical activity. Neuroscience and biobehavioral reviews. 2019;107:525-39. Epub 2019/10/06. doi: 10.1016/j.neubiorev.2019.09.040. PubMed PMID: 31586447.

30. Wray A, Martin G, Ostermeier E, Medeiros A, Little M, Reilly K, et al. Physical activity and social connectedness interventions in outdoor spaces among children and youth: a rapid review. Health promotion and chronic disease prevention in Canada : research, policy and practice. 2020;40(4):104-15. Epub 2020/04/10. doi: 10.24095/hpcdp.40.4.02. PubMed PMID: 32270668; PubMed Central PMCID: PMCPMC7197641.

31. Brussoni M, Olsen LL, Pike I, Sleet DA. Risky play and children's safety: balancing priorities for optimal child development. International journal of environmental research and public health. 2012;9(9):3134-48. Epub 2012/12/04. doi: 10.3390/ijerph9093134. PubMed PMID: 23202675; PubMed Central PMCID: PMCPMC3499858.

32. Thompson Coon J, Boddy K, Stein K, Whear R, Barton J, Depledge MH. Does participating in physical activity in outdoor natural environments have a greater effect on physical and mental wellbeing than physical activity indoors? A systematic review. Environmental science & technology. 2011;45(5):1761-72. Epub 2011/02/05. doi: 10.1021/es102947t. PubMed PMID: 21291246.

33. Ahola R, Pyky R, Jämsä T, Mäntysaari M, Koskimäki H, Ikäheimo TM, et al. Gamified physical activation of young men--a Multidisciplinary Population-Based Randomized Controlled Trial (MOPO study). BMC public health. 2013;13:32. Epub 2013/01/15. doi: 10.1186/1471-2458-13-32. PubMed PMID: 23311678; PubMed Central PMCID: PMCPMC3553029.

34. Jurado-Castro JM, Gil-Campos M, Llorente-Cantarero FJ. Methods recently used for the assessment of physical activity in children and adolescents. Current opinion in clinical nutrition and metabolic care. 2022;25(5):298-303. Epub 2022/07/06. doi: 10.1097/mco.0000000000000847. PubMed PMID: 35788094.

35. Ridgers ND, McNarry MA, Mackintosh KA. Feasibility and Effectiveness of Using Wearable Activity Trackers in Youth: A Systematic Review. JMIR mHealth and uHealth. 2016;4(4):e129. Epub 2016/11/25. doi: 10.2196/mhealth.6540. PubMed PMID: 27881359; PubMed Central PMCID: PMCPMC5143467.

36. Creaser AV, Clemes SA, Costa S, Hall J, Ridgers ND, Barber SE, et al. The Acceptability, Feasibility, and Effectiveness of Wearable Activity Trackers for Increasing Physical Activity in Children and Adolescents: A Systematic Review. International journal of environmental research and public health. 2021;18(12). Epub 2021/07/03. doi: 10.3390/ijerph18126211. PubMed PMID: 34201248; PubMed Central PMCID: PMCPMC8228417.

37. Ridgers ND, Timperio A, Brown H, Ball K, Macfarlane S, Lai SK, et al. Wearable Activity Tracker Use Among Australian Adolescents: Usability and Acceptability Study. JMIR mHealth and uHealth. 2018;6(4):e86. Epub 2018/04/13. doi: 10.2196/mhealth.9199. PubMed PMID: 29643054; PubMed Central PMCID: PMCPMC5917084.

38. Kellett S, Simmonds-Buckley M, Bliss P, Waller G. Effectiveness of Group Behavioural Activation for Depression: A Pilot Study. Behavioural and cognitive psychotherapy. 2017;45(4):401-18. Epub 2017/03/14. doi: 10.1017/s1352465816000540. PubMed PMID: 28287065.

39. Lejuez CW, Hopko DR, Acierno R, Daughters SB, Pagoto SL. Ten year revision of the brief behavioral activation treatment for depression: revised treatment manual. Behavior modification. 2011;35(2):111-61. Epub 2011/02/18. doi: 10.1177/0145445510390929. PubMed PMID: 21324944.

40. Montgomery SA, Asberg M. A new depression scale designed to be sensitive to change. The British journal of psychiatry : the journal of mental science. 1979;134:382-9. Epub 1979/04/01. doi: 10.1192/bjp.134.4.382. PubMed PMID: 444788.

41. Reliability and validity of the Chinese version of Beck Depression Inventory-II among depression patients [Internet]. China: Chinese Mental Health; 2011

42. Shudo Y, Yamamoto T. Assessing the relationship between quality of life and behavioral activation using the Japanese Behavioral Activation for Depression Scale-Short Form. PloS one. 2017;12(9):e0185221. Epub 2017/09/29. doi: 10.1371/journal.pone.0185221. PubMed PMID: 28957346; PubMed Central PMCID: PMCPMC5619729.

43. Yuhui W, Wan L, Jiahu H, Fangbiao T, Maternal DO, Childamp, et al. Development and evaluation on reliability and validity of Adolescent Non-suicidal Self-injury Assessment Questionnaire. 2018.

44. Morin CM, editor Insomnia: Psychological Assessment and Management1993.

45. Craig CL, Marshall AL, Sjöström M, Bauman AE, Booth ML, Ainsworth BE, et al. International physical activity questionnaire: 12-country reliability and validity. Medicine and science in sports and exercise. 2003;35(8):1381-95. Epub 2003/08/06. doi: 10.1249/01.Mss.0000078924.61453.Fb. PubMed PMID: 12900694.

46. Szuhany KL, Otto MW. Efficacy evaluation of exercise as an augmentation strategy to brief behavioral activation treatment for depression: a randomized pilot trial. Cognitive behaviour therapy. 2020;49(3):228-41. Epub 2019/07/31. doi: 10.1080/16506073.2019.1641145. PubMed PMID: 31357916; PubMed Central PMCID: PMCPMC6989384.
