# Supplementary material for: The effect of behavioral activation play therapy in adolescents with depression: A study protocol for a randomized controlled trial
Source: PLoS One. 2024 Jun 20;19(6):e0304084. doi: 10.1371/journal.pone.0304084 (PMC11189190; doi:10.1371/journal.pone.0304084)
Supplement: S2 File — Translated version of the original protocol approved by the Regional Committee for Medical and Health Research Ethics. (DOCX) [file pone.0304084.s002.docx]

**Study projects**:

## The effect of behavioral activation play therapy in adolescents with depression: a study protocol for a randomized controlled trial

Principal Investigator and Organizer:

Xiaolong Huang

Department of Child and Adolescent Psychiatry

The Affiliated Brain Hospital of Guangzhou Medical University

Guangzhou 510100, China

Tel: 86-20-81268002

Email: XiaoLongHuang0713@hotmail.com

Status: Mar. 17, 2022

**The effect of behavioral activation play therapy in adolescents with depression: a study protocol for a randomized controlled trial**

Study Group

This project is a collaboration between the Guangzhou Medical University and the Department of Child and Adolescent Psychiatry, the Affiliated Brain Hospital of Guangzhou Medical University.

Principal Investigator

Xiaolong Huang, Department of Child and Adolescent Psychiatry of the Affiliated Brain Hospital of Guangzhou Medical University, Guangzhou 510100, China. E-mail: [XiaoLongHuang0713@hotmail.com](mailto:XiaoLongHuang0713@hotmail.com), Tel: 86-20-81268002

Major scientists involved in the Probiotics Study

Xiaolong Huang, the Affiliated Brain Hospital of Guangzhou Medical University, Guangzhou, China

Yanling Zhou, the Affiliated Brain Hospital of Guangzhou Medical University, Guangzhou, China

Chanjuan Yang, the Affiliated Brain Hospital of Guangzhou Medical University, Guangzhou, China

Wei Luo, the Affiliated Brain Hospital of Guangzhou Medical University, Guangzhou, China

Dongdong Wang, the Affiliated Brain Hospital of Guangzhou Medical University, Guangzhou, China

Yuqi Chen, the Southern Medical University, Guangzhou, China

Jiacheng Luo, Guangzhou Medical University, Guangzhou, China

Synopsis

In this study, we integrated behavioral activation (BA) and gamified physical activities (GPA) to create behavioral activation play therapy (BAPT). This is the first RCT to explore the effectiveness and adaptability of BAPT in adolescents with depression. This study will provide evidence that may help to decrease depressive symptoms in adolescents, and will demonstrate the treatment effectiveness in terms of increasing levels of physical activity, reducing the rate of non-suicidal self-injury behaviors, and improving sleep quality. We also hope to assess other possible clinical effects of BAPT.

Participants aged 12 to 17 years will be eligible if they are defined as having depression by using DSM-5, but if they meet one of the following exclusion criteria they will not be allowed to take part: (1) Participants with a DSM-5 diagnosis of other mental disorders, including addictive disorders, developmental disorders, bipolar disorder, substance-related disorders, and schizophrenia (except anxiety disorders); (2) Participants with severely disruptive or aggressive behaviors, or positive suicidal ideation (suicide item scores of the MADRS > 4); (3) Participants with severe psychotic symptoms (presence of pain or common hallucinations and/or delusions); (4) Participants with clinically significant and uncontrolled pulmonary, endocrine, immunological, cardiovascular diseases (based on ancillary examination, physical examination, medical history); (5) According to the research group, participants who could not cooperate with the cognitive function tests or would not be suitable for this study.

Prior to participation in this study, all subjects and their legal guardians must be fully informed about the project and sign a written informed consent form. The participants will be divided into intervention group and control group according to a pre-defined randomization protocol. The intervention and control groups received 4 weeks of BAPT and 4 weeks of BA respectively. Participants in both groups will be treated and cared for according to the same criteria, but will not be involved in any other psychological therapy programs.

Therapeutic effect evaluation will be done at the baseline, week 2 (the end of the basic intervention), week 4 (the end of all the high-order intervention), week 8, and week 16 by the MADRS, BDI-II-C, BADS-SF, ANSAQ, ISI, IPAQ-SF, and Likert Scale. The primary outcome was whether BAPT could decrease depressive symptoms in adolescents by MADRS scores.

# Background

## Epidemiology

From 2012 to 2022, the incidence of depression in adolescents increased dramatically[1]. In 2020, 17.2% and 7.4% of adolescents in China were found to have mild and severe depressive symptoms, respectively, and the lifetime prevalence is expected to be 11%–20%[2]. The high prevalence of depression in adolescents is a public health issue because it can interrupt the developmental process and have negative effects throughout life[3, 4]. Adolescent major depressive disorder (MDD) can have serious consequences such as dropping out of school, drug use, self-harm, and suicide, and it is the main cause of illness and disability among adolescents. Currently, clinical guidelines recommend psychotherapy and medication for adolescents with depression[5]. However, psychotherapy is associated with a number of drawbacks, as it can often be difficult to access, costly, and minimally effective. Furthermore, some medications prescribed to treat adolescent MDD, such as fluoxetine and venlafaxine, have unfortunate side effects including increasing suicidal thoughts and behaviors in adolescents. As a result of these drawbacks, some adolescents with depression do not benefit from medication and psychotherapies[6]. Accordingly, effective interventions are an important focus for mental health researchers around the world[7, 8].

## Physical activity

In recent years, exercise therapy has attracted worldwide attention because of its simplicity and strong effects[9]. For instance, the effects of high and moderate intensity exercise have been shown to be comparable to those of antidepressant treatment[10]. Consequently, clinicians have adopted an increasingly positive attitude towards the use of exercise in treating adolescents with MDD[11]. In 2020, the World Health Organization (WHO) recommended that children and adolescents engage in at least one hour of moderate-to-high physical activity (PA) every day to improve health and reduce symptoms of depression[12]. However, research showed that only 20% of children and adolescents aged 13–15 comply with this recommendation[13]. Adolescents with depression are even less likely to engage in exercise, as depressive symptoms reduce the motivation to exercise[14]. Therefore, more effective treatment strategies are urgently needed.

## Behavioral activation

In 2023, the WHO recommended behavioral activation (BA) as an effective form of psychotherapy for the treatment of depression and suggested that antidepressants should not be used as first-line treatment for adolescents[15]. BA is clinically as effective as cognitive behavioral therapy (CBT), costs up to 21% less[16], and is simple and easy to administer. Although BA is an effective treatment for adolescents with depression, it is difficult to conduct in the adolescent population and outcomes have been found to be insufficient[17]. As a result, health practitioners have attempted to apply BA in combination with other therapeutic techniques to obtain better treatment results in adolescents with depression[18-20]. For example, the combination of BA with digital networking technology was well received by adolescents[21, 22], but this program led to an increase in screen time. Besides, increased screen time in adolescents may lead to a decrease in social and physical activity as well as an increase in sedentary behavior[23]. Furthermore, the depression risk increases in individuals who exceed two hours of screen time per day[24].

## BA combined with PA

BA including PA can improve mood, which is beneficial for patients with depression, along with increasing the amount of regular exercise[16]. Studies have shown that BA interventions combined with PA have a high completion rate, and that they can significantly reduce depressive symptoms in patients. BA and PA are highly consistent in terms of techniques and methods, such as self-monitoring, goal setting, and problem-solving techniques. Combining them could overcome the limitations of using each intervention alone, as well as reduce the recurrence rate of depression after treatment[25].

Although studies have demonstrated that BA combined with PA is feasible and acceptable for improving depressive symptoms in adults, fewer studies have been conducted in adolescents. This may be because different age groups choose different forms of PA. In contrast to traditional forms of PA, adolescents are more inclined to select game forms of PA[26]. Therefore, it is crucial to increase the degree to which physical activities are perceived as pleasurable when developing therapies for use in this population[27]. Studies have shown that using gamification to promote physical activity can lead to better uptake[28], indicating that interventions that focus on gamified physical activity (GPA) may be a practical way to reduce sedentary behavior and depression risk in adolescents[29]. In most adolescents, play is a vital element of PA interventions, and the integration of play-centered activities is likely to be an effective method for increasing the level of PA and improving social connections. Indeed, encouraging adolescents to participate in active, stimulating, and adventurous play could enable them to test their abilities independently, thus improving social resilience[30-32]. Using competition and rewards may help to promote physical activity, along with other more innovative approaches[33].

## Study objectives

To date, no empirical studies have verified the effects of interventions that integrate GPA into BA with the goal of improving depressive symptoms in adolescents with depression in clinical settings in China. As a result, there are no guidelines regarding the parameters of exercise necessary for anti-depressive effects. To address this, our research group integrated BA and GPA into behavioral activation play therapy (BAPT), and developed a randomized controlled trial (RCT) to evaluate the efficacy of BAPT for treating adolescents with depression. We plan to assess the effectiveness of BAPT in increasing levels of physical activity, reducing non-suicidal self-injury behavior, and ameliorating sleep quality. We also hope to discern the adaptability of this treatment in terms of clinical applications.

## Hypotheses

Primary: BAPT (intervention group) will be more effective than BA (control group) in relieving depressive symptoms and enhancing treatment adherence in adolescents with depression.

Secondary: BAPT (intervention group) will be more effective than BA (control group) in increasing levels of physical activity, reducing non-suicidal self-injury behavior, and ameliorating sleep quality.

# Methods and design

## Study design and setting

This study is a two-arm single-blind RCT. The intervention and control groups will receive 9 sessions of BAPT and 9 sessions of BA, respectively. Because adolescents with depression who are hospitalized generally have a relatively short stay in the hospital, the participants in this study will complete the first 4 sessions during hospitalization and then will be asked to return to the department for the remaining 5 sessions after they are discharged.

This study was approved by the ethics committee of the Affiliated Brain Hospital of Guangzhou Medical University on April 27, 2023 (Ethics number: 2023 [027]) and registered with the China Clinical Trials Center under the registration number ChiCTR2300072671. The design of this protocol is in line with the Standard Protocol Items: Recommendations for Intervention Trials (SPIRIT). **Fig. 1** presents the enrolment, intervention and evaluation schedule for SPIRIT. **Fig. 2** presents a flow chart of the study. The study will be supervised by the Department of Child and Adolescent Psychiatry (DCAP) and the Academic Management Committee of the Affiliated Brain Hospital of Guangzhou Medical University.

### Fig. 1 Study period


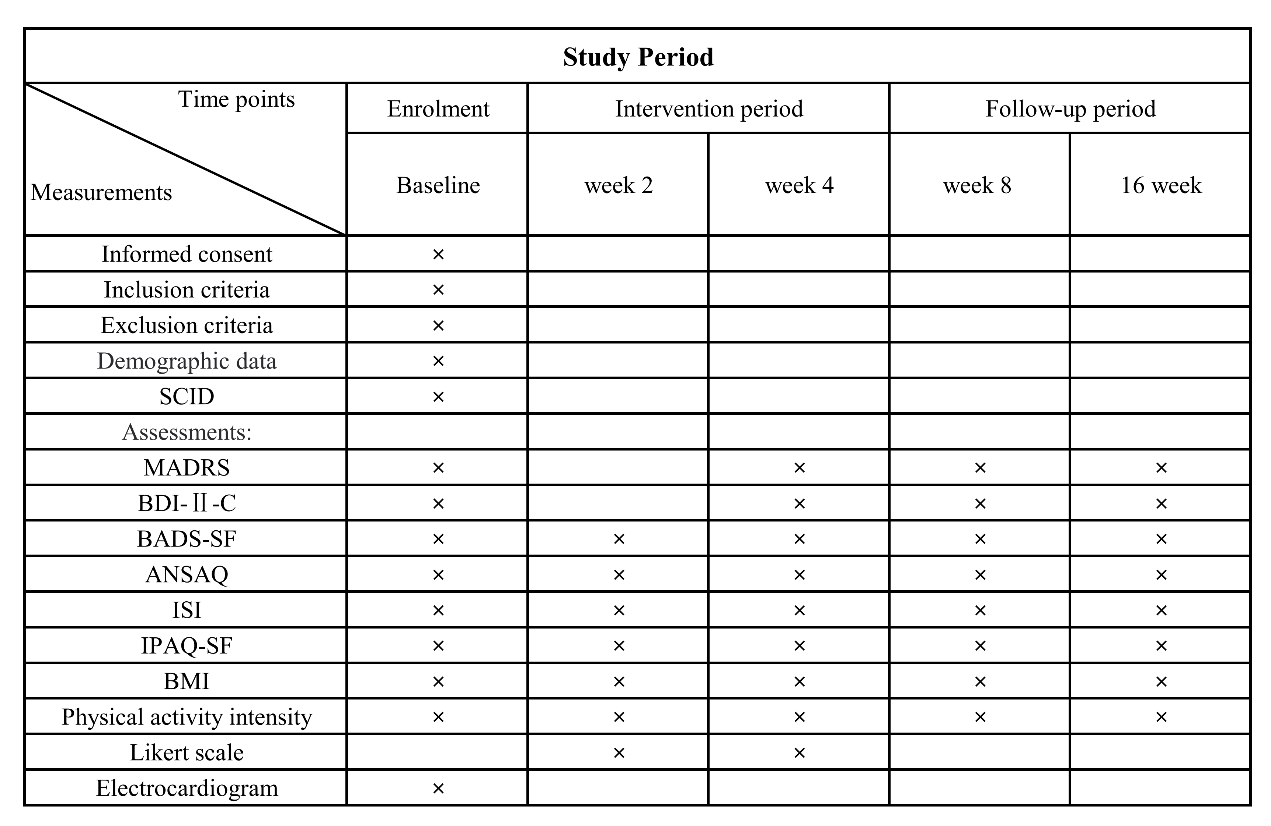


**Abbreviations:** ***BMI***: Body Mass Index; ***MADRS***: Montomery-Asberg Depression Rating Scale; ***BDI-Ⅱ-C***: Beck Depression Inventory-China; ***BADS-SF***: Behavioral Activation for Depression Scale Short Form; ***ANSAQ***: Adolescent Non-suicidal Self-injury Assessment Questionnaire; ***ISI***: Insomnia Severity Index; ***IPAQ-SF***: International Physical Activity Scale Short Form; ***Physical activity intensity***: collecting data regarding calorie consumption (kcal) and metabolic equivalents; ***Likert Scale***: assessing the acceptance of the intervention by patients and their families.

### Fig. 2 Research flow chart


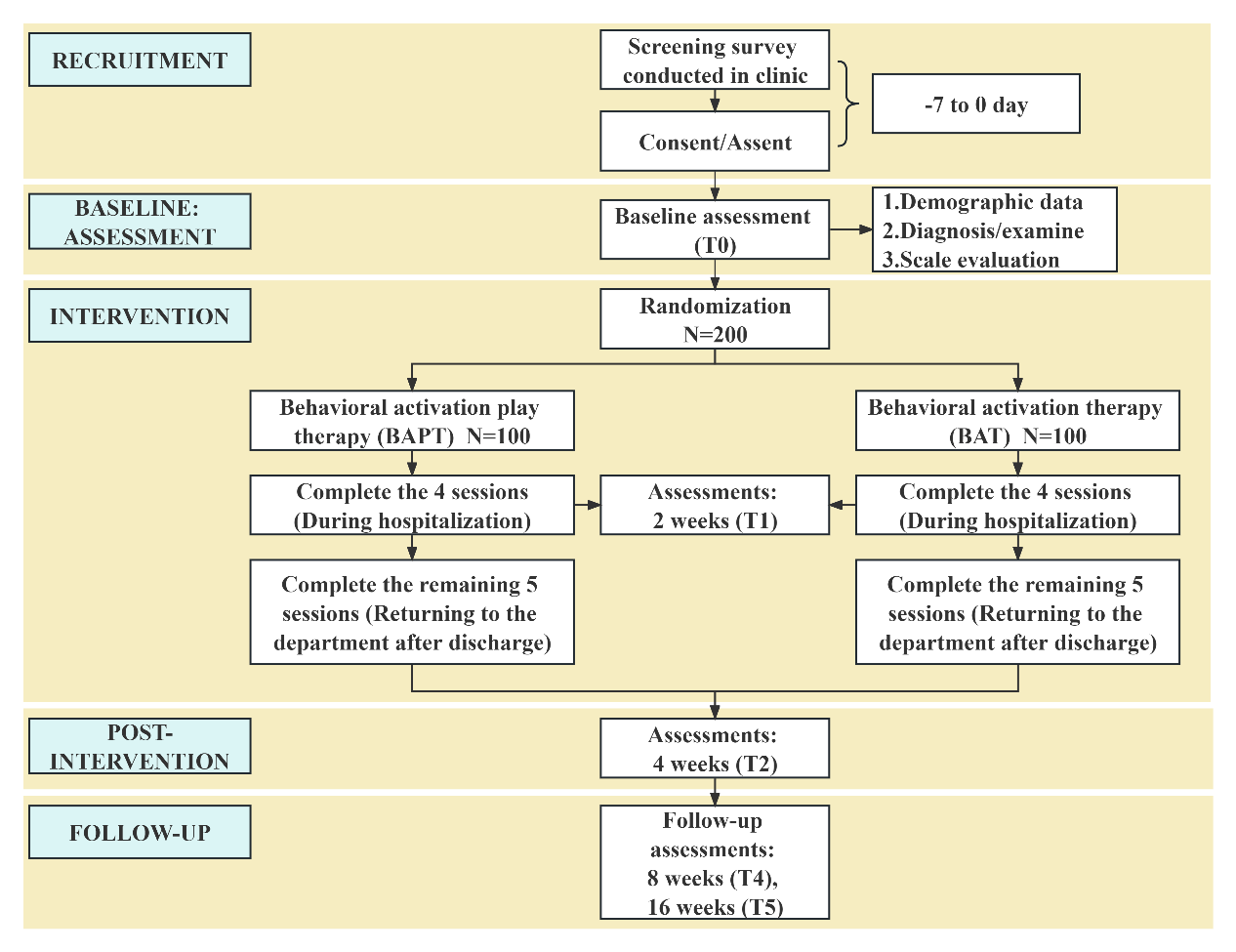


*****Participant data regarding physical intensity were collected every week during the study.

## Recruitment

Participants will be recruited at the Department of Child and Adolescent Psychiatry in the Affiliated Brain Hospital of Guangzhou Medical University. Prior to screening, researchers will offer an explanation of the study process to the participants and their legal guardians, along with the significance of the study, risks, and benefits. All participants and their legal guardians will to partake the study voluntarily, as indicated by signing an informed consent form, and will be able to withdraw at any time in the process of the study. The interventions provided in the study will be free of charge.

## Participant selection

Participants were diagnosed with depression based on the DSM-5 by two senior experts in the same field. Listed in **Table 1** are the inclusion and exclusion criteria for adolescents with depression.

### Table 1 Inclusion and exclusion criteria

| **Subject eligibility** | |
| --- | --- |
| **Inclusion**  **criteria**  **Exclusion criteria** | 1. Met the criteria for a DSM-5 diagnosis of depression without psychotic features; 2. MADRS scores ≥ 12; 3. Inpatients aged 12–17 years; 4. Participants with intelligence level in the normal range, normal perception, self-expression ability, and who can complete the baseline scale assessment; 5. Informed consent signed by the participants and their legal guardians. 6. Participants with a DSM-5 diagnosis of other mental disorders, including addictive disorders, developmental disorders, bipolar disorder, substance-related disorders, and schizophrenia (except anxiety disorders); 7. Participants with severely disruptive or aggressive behaviors, or positive suicidal ideation (suicide item scores of the MADRS > 4); 8. Participants with severe psychotic symptoms (presence of pain or common hallucinations and/or delusions); 9. Participants with clinically significant and uncontrolled pulmonary, endocrine, immunological, cardiovascular diseases (based on ancillary examination, physical examination, medical history); 10. According to the research group, participants who could not cooperate with the cognitive function tests or would not be suitable for this study. |

**Abbreviations**: ***DSM-5***: Diagnostic and Statistical Manual of Mental Disorders; ***MADRS***: Montgomery-Asberg Depression Rating Scale.

## Randomization

Random sequences will be generated by a specially recruited staff member, who will use the SPSS Rv.Uniform function to generate a random number table. The random seed parameters and groupings will be sealed as confidential data in an opaque envelope. The random sequence and grouping data will be kept by one designated person, who will open the envelope after the subjects meeting the criteria are selected to enter the study and have signed the informed consent form. The investigator will be informed regarding the participants’ treatment allocation, and the participants will be randomized according to a pre-defined randomization protocol.

## Blinding

This study will use a single-blind approach because in this intervention, the participants and health care professionals cannot be blinded to the study conditions. Therefore, to minimize the impact of information bias, the researcher administering the outcome measurement will be unaware of the group assignment. To maintain researcher blindness regarding group assignment, the same researcher will not conduct follow-up measurements on the same pair of elements.

## Intervention

All interventions will be conducted in a group format and delivered by experienced psychotherapists. All participants will use electronic exercise wristbands (Beijing Xiaomi Technology Co., LTD., [Product number](javascript:;): Xiaomi Band 7pro, M2140B1) to record the daily intensity of their physical activities. The electronic exercise wristbands utilize photocapacitance pulse wave tracing (PPG) and acceleration sensors to measure the intensity of physical activity in adolescents[34-37]. During the study period, the participants will only receive conventional clinical medication and care, and will not be involved in any other psychological therapy programs.

## Intervention group: BAPT

The intervention group will receive 9 BAPT sessions, twice a week, for 60 minutes each time. Each session will consist of BA (30 minutes) and GPA (30 minutes). The BA procedure was adapted from the protocols of Kellett et al.[38] and Lejuez et al.[39], with appropriate modifications according to the actual situation. In the BA sessions, participants will receive information about the core principles of BA, which can be summarized as nine topics as follows: (1) Initiation of BA: the sense of values and connection; (2) the BA model and emotional monitoring; (3) getting motivated: goal-oriented behavior; (4) getting activated: situation-action-emotion; (5) problem solving skills; (6) goal setting and adjustment; (7) identifying obstacles and overcoming avoidance; (8) thinking: worrying, ruminating; (9) prevention of recurrence. Each session will contain one topic, and the GPA associated with that topic will be completed after that part of the BA. The BAPT scheme is shown in **Table 2**. All GPAs were designed or modified by the research team to be competitive, adventurous, interactive, and to follow certain rules that are in line with the physical and mental development characteristics of adolescents[26]. For example, “Cat catches mouse” is a type of exciting chase game in which participants are asked to choose one of three roles: one pair of participants are the cat and mouse, while the others are fences. All of the fences form a circle such that the participants are standing about 1 meter apart. The mouse is inside the circle and the cat is outside, and when the game starts, the cat attempts to enter the circle to catch the mouse. To avoid being captured, the mouse can choose a fence by touching that participant. Then, the original fence becomes the cat, the original cat becomes a mouse, and the cycle continues until the cat catches the mouse. The game is interesting and challenging because of the constantly changing roles of the participants in the game. The researchers rated all GPAs as moderate to high intensity physical activity after testing with electronic exercise wristbands. Therefore, we have mapped out a complete risk prevention scenario designed to protect the safety of the participants. If an adverse event occurs, it will be recorded and reported to the Ethics Committee.

After each session, the participants will receive homework, where they will be encouraged to schedule activities (socio-motor game, etc.) that feel enjoyable, rewarding, and offer a sense of control during the following week, and to monitor the impact of these activities on their personal mood. Homework will be collected by the researchers once a week.

### Table 2 The scheme of the BAPT

| Time | Session | Course theory | Game-type physical activity |
| --- | --- | --- | --- |
| Weeks 1 - 2  (During hospitalization) | 1 | Initiate BA: the sense of values and connection | Strong Winds Blow |
|  | 2 | The BA model and emotional monitoring | Balloon Missile |
|  | 3 | Get motivated: goal-oriented behavior | Giant's Hat |
|  | 4 | Get activated: situation-action-emotion | Overcome The Obstacle |
| Weeks 3 - 4  (Returning to the  department after  discharge) | 5 | Problem solving skills | Transmitting Things By Sole |
|  | 6 | Goal setting and adjustment | Blindfolded Communication |
|  | 7 | Identify obstacles and overcome avoidance | Cat Catches Mouse |
|  | 8 | Thinking: worrying, ruminating | Knee Pat |
|  | 9 | Prevention of recurrence | Queen Ant Game |

**Abbreviations:** Weeks 1–2: complete sessions 1–4; Weeks 3–4: complete sessions 5–9.

## Control group: BA

The control group intervention will be same as the BA in the intervention group, but without the GPA. Each session will be 60 minutes long because of the extended discussion. Homework will be the same as in the intervention group, but without the socio-motor game suggestions.

## Intervention Fidelity

The BA or BAPT interventions will be delivered by psychotherapists recruited from the clinical staff of the Affiliated Brain Hospital of Guangzhou Medical University. All psychotherapists will complete a two-week consistency training program. Group supervision will be conducted by a senior clinical psychologist throughout the study, and all sessions will be recorded so that the treatment adherence can be assessed.

## Safety and monitoring

Before each intervention session, the subjects will complete the 10th item of the clinician-rated Montgomery-Asberg Depression Rating Scale (MADRS) for suicidal risk. If the scores are > 4, the subjects will be temporarily withdrawn from the study. During the intervention, if the subjects show severe self-injury or suicidal behavior, they also will be temporarily withdrawn from the study. Subsequently, the doctors in the project team will conduct suicide crisis interventions and follow-up visits, and then organize expert discussions regarding any incidents to avoid recurrence.

## Efficacy evaluations

From week 0 to week 16, researchers will utilize MADRS scores as a primary outcome to assess changes in depressive symptoms. A total score of 0–60 is obtained by completing 10 items with scores ranging from 0 to 6[40]. The depressive symptoms will be grouped as extreme, major, moderate, or mild according to the following scores: MADRS > 35, 30 ≤ MADRS < 35, 22 ≤ MADRS < 30, 12 ≤ MADRS < 22, respectively, and MADRS < 12 indicates no depressive symptoms. Compared with the baseline, a MADRS total score reduction of 50% will be defined as a significant antidepressant response, while a reduction of 20% will be defined as an improvement.

Secondary outcome measures include: (1) Self-rated depressive symptoms on the Baker Depression Scale, second Chinese version (BDI-II-C): This scale has high internal consistency, and reflects the severity of depression in the past two weeks. It contains 21 items, each rated on a 0–3 scale, with an overall score of 0–63 points. The levels of depression will be grouped as severe, moderate, or mild according to the following ranges of scores: 29–63, 20–28, and 14–19, respectively, and a score of 0–13 will be classified as no depression[41]. (2) Behavioral Activation for Depression Scale Short Form (BADS-SF): This scale consists of nine items that measure changes in behavioral activation during the past week, including the measurement day. The activation (AC) subscale includes questions 1, 2, 3, 4, 5, and 9, while the avoidance (AV) subscale includes questions 6, 7, and 8. The scale uses a 7-level scoring method, which ranges from 0 (not at all) to 6 (completely). A higher score for an item represents an answer that is closer to the item statement. The scale has been demonstrated to have robust reliability and validity[42] (3) Adolescent Non-suicidal Self-injury Assessment Questionnaire (ANSAQ): This questionnaire is divided into two parts: a behavioral questionnaire (12 items) and a functional questionnaire (19 items) to evaluate self-injury behavior[43]. It uses the Likert 5-point scale, where “1 to 5” corresponds with “no, occasionally, sometimes, often, always”, respectively. A higher score represents a more serious degree of self-injury. The questionnaire has high internal consistency, while that of the behavioral questionnaire was 0.921. In this study, we will only use the behavioral dimension of the questionnaire (12 questions in total) to assess whether the depressed adolescents engage in self-injury behavior. (4) Insomnia Severity Index (ISI): this is used to evaluate the severity of insomnia in patients. Total scores of 0–28 are obtained according to responses to 7 items with scores ranging from 0 to 4[44]. According to the scoring guide, scores for mild, moderate, and severe clinical insomnia range from 8–14, 15–21, and 22–28, respectively, while scores less than 7 are considered to represent non-clinical insomnia. (5) International Physical Activity Questionnaire-Short Forms (IPAQ-SF): This short form records activity for four intensity levels: 1) vigorous-intensity activity such as aerobics, 2) moderate-intensity activity such as leisure cycling, 3) walking, and 4) sitting[45]. These four intensity levels (except sitting) can be determined and reported as MET-minutes per week. The IPAQ-SF has good internal consistency. (6) Custom 5-point Likert score acceptance questionnaire: to assess the acceptance of the intervention by participants and their families.

At the baseline, week 2 (the end of the basic intervention), week 4 (the end of all the high-order intervention), week 8, and week 16, participants from the two groups will be evaluated with the corresponding scales to assess the degree of depression, behavioral activation level, non-suicidal self-injury behavior, and sleep quality scores. We will also calculate the improvement of symptoms for each dimension in each group at each time point, and then compare the divergences between the two groups. All participants will receive an electronic exercise wristband as a reward after they completed the follow-up.

## Demographic and clinical data

Demographic data (age, sex, occupation, culture, residence, family structure, economic income, etc.), medical expenses, growth history, history of tobacco, alcohol and other psychoactive substance use, psychiatric symptoms, and length of hospital stay will be collected during the screening stage. All participants and their families will volunteer to participate and sign informed consent.

## Auxiliary inspection:

Electrocardiograms, routine blood analysis, and biochemical indexes of clinical treatment will be completed at the baseline assessment.

## Physical activity intensity

The Metabolic Equivalent (MET) can be used to express the relative energy metabolism level of physical activity. Depending on the intensity of the exercise, which can be divided into light, medium, or heavy grade, it can be represented by a score of 0–3, 3–6, and more than 6, respectively.

The researchers will collect data regarding the participants' physical activity intensity once a week. Participants who complete the homework and exercise at a moderate intensity for 150 minutes or above per week will be rewarded with small gifts for activation and positive reinforcement. The electronic exercise wristbands will be purchased and distributed by the project team to ensure monitoring consistency.

## Data management

The Case Report Form (CRF) will be utilized to document the demographic information and clinical symptoms of all participants, which will subsequently be stored in the database through a process of double data entry. The primary responsibility of the study leader will be to ensure the integrity, accuracy, and promptness of the data inputting process. In an effort not to disclose the privacy of participants, researchers will remove the names, phone numbers, and addresses that are not related to the study. Numbers will be used to identify the participants. In addition, the original CRF will be securely stored throughout the study and will be accessible to the project leader. A dedicated physician (Prof. Yanling Zhou) will monitor the wholeness, uniformity and plausibility of the data.

## Statistical analysis

### Sample size calculation

The depressive symptom scores of the study subjects will be used as the main outcome index, with a bilateral α = 0.05 and a 90% confidence interval. In a previous study on the effects of BA combined with traditional exercise therapy, the depression scores of the intervention group at the baseline and at the 16-week follow-up period were 27.3 ± 8.0 and 16.0 ± 9.5, respectively[46]. Therefore, we expect that the depression score of the experimental group in the present study could be decreased by 11.3 points compared with the baseline and 4.8 points compared with the control group. We used PASS 15 software to calculate the sample size of the experimental group: N1 = N2 = 84. Considering the expectation that 10%­–20% of the cases will be

lost during the period following the study, the number of participants needed in the intervention group and control group will be about 100, respectively.

## Data analysis

The study is based on the intention-to-treat principle. SPSS 28.0 will be used for data analysis. First, to ensure comparability after randomization, we will compare the differences between the groups at the baseline. The continuous variables will be analyzed by a t-test when the data have a normal distribution, and non-normally distributed data will be analyzed using the Mann-Whitney U test. Baseline differences between groups in terms of sociodemographic and clinical variables will be assessed using Chi-square tests for categorical variables and an analysis of variance (ANOVA) for continuous data. To compare intervention effects, a mixed-effect regression model will be used. Group (BAPT or BA) will be used as an inter-subject factor, and time (baseline, post-treatment, and follow-up) as an intra-subject factor. We will utilize Cohen's d to achieve both the intra- and intergroup analyses. P < 0.05 was set as statistically significant.

Financial Support

This study was supported by Science and Technology Program of Guangzhou (grant No. 205171098044), Guangzhou Municipal Key Discipline in Medicine (2021-2023), Guangzhou High-level Clinical Key Specialty, and Guangzhou Research-oriented Hospital.

## References

1. Thapar A, Eyre O, Patel V, Brent D. Depression in young people. The Lancet. 2022;400(10352):617-31. doi: <https://doi.org/10.1016/S0140-6736(22)01012-1>.

2. Press BSSA. China National Mental Health Development Report (2019-2020) Blue Book2021.

3. Clayborne ZM, Varin M, Colman I. Systematic Review and Meta-Analysis: Adolescent Depression and Long-Term Psychosocial Outcomes. Journal of the American Academy of Child and Adolescent Psychiatry. 2019;58(1):72-9. Epub 2018/12/24. doi: 10.1016/j.jaac.2018.07.896. PubMed PMID: 30577941.

4. Goldstein BI, Korczak DJ. Links Between Child and Adolescent Psychiatric Disorders and Cardiovascular Risk. The Canadian journal of cardiology. 2020;36(9):1394-405. Epub 2020/07/07. doi: 10.1016/j.cjca.2020.06.023. PubMed PMID: 32628978.

5. Summary of the clinical practice guideline for the treatment of depression across three age cohorts. The American psychologist. 2022;77(6):770-80. Epub 2021/11/30. doi: 10.1037/amp0000904. PubMed PMID: 34843274.

6. Oberste M, Medele M, Javelle F, Lioba Wunram H, Walter D, Bloch W, et al. Physical Activity for the Treatment of Adolescent Depression: A Systematic Review and Meta-Analysis. Frontiers in physiology. 2020;11:185. Epub 2020/04/09. doi: 10.3389/fphys.2020.00185. PubMed PMID: 32265725; PubMed Central PMCID: PMCPMC7096373.

7. Patton GC, Coffey C, Romaniuk H, Mackinnon A, Carlin JB, Degenhardt L, et al. The prognosis of common mental disorders in adolescents: a 14-year prospective cohort study. Lancet (London, England). 2014;383(9926):1404-11. Epub 2014/01/21. doi: 10.1016/s0140-6736(13)62116-9. PubMed PMID: 24439298.

8. Thapar A, Collishaw S, Pine DS, Thapar AK. Depression in adolescence. Lancet (London, England). 2012;379(9820):1056-67. Epub 2012/02/07. doi: 10.1016/s0140-6736(11)60871-4. PubMed PMID: 22305766; PubMed Central PMCID: PMCPMC3488279.

9. Zhang Y, Li G, Liu C, Guan J, Zhang Y, Shi Z. Comparing the efficacy of different types of exercise for the treatment and prevention of depression in youths: a systematic review and network meta-analysis. Frontiers in psychiatry. 2023;14:1199510. Epub 2023/06/19. doi: 10.3389/fpsyt.2023.1199510. PubMed PMID: 37333923; PubMed Central PMCID: PMCPMC10272399.

10. Balchin R, Linde J, Blackhurst D, Rauch HL, Schönbächler G. Sweating away depression? The impact of intensive exercise on depression. Journal of affective disorders. 2016;200:218-21. Epub 2016/05/04. doi: 10.1016/j.jad.2016.04.030. PubMed PMID: 27137088.

11. Radovic S, Melvin GA, Gordon MS. Clinician perspectives and practices regarding the use of exercise in the treatment of adolescent depression. Journal of sports sciences. 2018;36(12):1371-7. Epub 2017/09/26. doi: 10.1080/02640414.2017.1383622. PubMed PMID: 28945524.

12. WHO Guidelines Approved by the Guidelines Review Committee. WHO Guidelines on Physical Activity and Sedentary Behaviour. Geneva: World Health Organization

© World Health Organization 2020.; 2020.

13. Hallal PC, Andersen LB, Bull FC, Guthold R, Haskell W, Ekelund U. Global physical activity levels: surveillance progress, pitfalls, and prospects. Lancet (London, England). 2012;380(9838):247-57. Epub 2012/07/24. doi: 10.1016/s0140-6736(12)60646-1. PubMed PMID: 22818937.

14. Wang DQ, Zhang JJ, Chen JN, Li RY, Luo YX, Deng W. Exergames improves cognitive functions in adolescents with depression: study protocol of a prospective, assessor-blind, randomized controlled trial. BMC psychiatry. 2023;23(1):507. Epub 2023/07/14. doi: 10.1186/s12888-023-04967-7. PubMed PMID: 37442973; PubMed Central PMCID: PMCPMC10339627.

15. WHO. Depressive disorder (depression) 2023. Available from: <https://www.who.int/news-room/fact-sheets/detail/depression>.

16. Pass L, Lejuez CW, Reynolds S. Brief Behavioural Activation (Brief BA) for Adolescent Depression: A Pilot Study. Behavioural and cognitive psychotherapy. 2018;46(2):182-94. Epub 2017/08/02. doi: 10.1017/s1352465817000443. PubMed PMID: 28756787.

17. Cuijpers P, Karyotaki E, Ciharova M, Miguel C, Noma H, Stikkelbroek Y, et al. The effects of psychological treatments of depression in children and adolescents on response, reliable change, and deterioration: a systematic review and meta-analysis. European child & adolescent psychiatry. 2023;32(1):177-92. Epub 2021/10/07. doi: 10.1007/s00787-021-01884-6. PubMed PMID: 34611729; PubMed Central PMCID: PMCPMC9908674.

18. Grudin R, Ahlen J, Mataix-Cols D, Lenhard F, Henje E, Månsson C, et al. Therapist-guided and self-guided internet-delivered behavioural activation for adolescents with depression: a randomised feasibility trial. BMJ open. 2022;12(12):e066357. Epub 2022/12/27. doi: 10.1136/bmjopen-2022-066357. PubMed PMID: 36572500; PubMed Central PMCID: PMCPMC9806095.

19. Davidson TM, Yuen EK, Felton JW, McCauley J, Gros KS, Ruggiero KJ. Feasibility assessment of a brief, web-based behavioral activation intervention for adolescents with depressed mood. International journal of psychiatry in medicine. 2014;48(1):69-82. Epub 2014/10/31. doi: 10.2190/PM.48.1.f. PubMed PMID: 25354927.

20. Van Voorhees BW, Watson N, Bridges JF, Fogel J, Galas J, Kramer C, et al. Development and pilot study of a marketing strategy for primary care/internet-based depression prevention intervention for adolescents (the CATCH-IT intervention). Primary care companion to the Journal of clinical psychiatry. 2010;12(3). Epub 2010/10/15. doi: 10.4088/PCC.09m00791blu. PubMed PMID: 20944776; PubMed Central PMCID: PMCPMC2947535.

21. Bhattacharya A, Nagar R, Jenness J, Munson SA, Kientz JA. Designing Asynchronous Remote Support for Behavioral Activation in Teenagers With Depression: Formative Study. JMIR formative research. 2021;5(7):e20969. Epub 2021/07/14. doi: 10.2196/20969. PubMed PMID: 34255665; PubMed Central PMCID: PMCPMC8317030.

22. Grudin R, Vigerland S, Ahlen J, Widström H, Unger I, Serlachius E, et al. "Therapy without a therapist?" The experiences of adolescents and their parents of online behavioural activation for depression with and without therapist support. European child & adolescent psychiatry. 2023:1-10. Epub 2023/01/18. doi: 10.1007/s00787-023-02142-7. PubMed PMID: 36650254; PubMed Central PMCID: PMCPMC9844942.

23. Nakshine VS, Thute P, Khatib MN, Sarkar B. Increased Screen Time as a Cause of Declining Physical, Psychological Health, and Sleep Patterns: A Literary Review. Cureus. 2022;14(10):e30051. Epub 2022/11/17. doi: 10.7759/cureus.30051. PubMed PMID: 36381869; PubMed Central PMCID: PMCPMC9638701.

24. Liu M, Wu L, Yao S. Dose-response association of screen time-based sedentary behaviour in children and adolescents and depression: a meta-analysis of observational studies. British journal of sports medicine. 2016;50(20):1252-8. Epub 2015/11/11. doi: 10.1136/bjsports-2015-095084. PubMed PMID: 26552416; PubMed Central PMCID: PMCPMC4977203.

25. Farrand P, Pentecost C, Greaves C, Taylor RS, Warren F, Green C, et al. A written self-help intervention for depressed adults comparing behavioural activation combined with physical activity promotion with a self-help intervention based upon behavioural activation alone: study protocol for a parallel group pilot randomised controlled trial (BAcPAc). Trials. 2014;15:196. Epub 2014/06/03. doi: 10.1186/1745-6215-15-196. PubMed PMID: 24886116; PubMed Central PMCID: PMCPMC4061537.

26. Nijhof SL, Vinkers CH, van Geelen SM, Duijff SN, Achterberg EJM, van der Net J, et al. Healthy play, better coping: The importance of play for the development of children in health and disease. Neuroscience and biobehavioral reviews. 2018;95:421-9. Epub 2018/10/03. doi: 10.1016/j.neubiorev.2018.09.024. PubMed PMID: 30273634.

27. Kagawa F, Yokoyama S, Takamura M, Takagaki K, Mitsuyama Y, Shimizu A, et al. Decreased physical activity with subjective pleasure is associated with avoidance behaviors. Scientific reports. 2022;12(1):2832. Epub 2022/02/20. doi: 10.1038/s41598-022-06563-3. PubMed PMID: 35181696; PubMed Central PMCID: PMCPMC8857298.

28. Mazeas A, Duclos M, Pereira B, Chalabaev A. Evaluating the Effectiveness of Gamification on Physical Activity: Systematic Review and Meta-analysis of Randomized Controlled Trials. Journal of medical Internet research. 2022;24(1):e26779. Epub 2022/01/05. doi: 10.2196/26779. PubMed PMID: 34982715; PubMed Central PMCID: PMCPMC8767479.

29. Kandola A, Ashdown-Franks G, Hendrikse J, Sabiston CM, Stubbs B. Physical activity and depression: Towards understanding the antidepressant mechanisms of physical activity. Neuroscience and biobehavioral reviews. 2019;107:525-39. Epub 2019/10/06. doi: 10.1016/j.neubiorev.2019.09.040. PubMed PMID: 31586447.

30. Wray A, Martin G, Ostermeier E, Medeiros A, Little M, Reilly K, et al. Physical activity and social connectedness interventions in outdoor spaces among children and youth: a rapid review. Health promotion and chronic disease prevention in Canada : research, policy and practice. 2020;40(4):104-15. Epub 2020/04/10. doi: 10.24095/hpcdp.40.4.02. PubMed PMID: 32270668; PubMed Central PMCID: PMCPMC7197641.

31. Brussoni M, Olsen LL, Pike I, Sleet DA. Risky play and children's safety: balancing priorities for optimal child development. International journal of environmental research and public health. 2012;9(9):3134-48. Epub 2012/12/04. doi: 10.3390/ijerph9093134. PubMed PMID: 23202675; PubMed Central PMCID: PMCPMC3499858.

32. Thompson Coon J, Boddy K, Stein K, Whear R, Barton J, Depledge MH. Does participating in physical activity in outdoor natural environments have a greater effect on physical and mental wellbeing than physical activity indoors? A systematic review. Environmental science & technology. 2011;45(5):1761-72. Epub 2011/02/05. doi: 10.1021/es102947t. PubMed PMID: 21291246.

33. Ahola R, Pyky R, Jämsä T, Mäntysaari M, Koskimäki H, Ikäheimo TM, et al. Gamified physical activation of young men--a Multidisciplinary Population-Based Randomized Controlled Trial (MOPO study). BMC public health. 2013;13:32. Epub 2013/01/15. doi: 10.1186/1471-2458-13-32. PubMed PMID: 23311678; PubMed Central PMCID: PMCPMC3553029.

34. Jurado-Castro JM, Gil-Campos M, Llorente-Cantarero FJ. Methods recently used for the assessment of physical activity in children and adolescents. Current opinion in clinical nutrition and metabolic care. 2022;25(5):298-303. Epub 2022/07/06. doi: 10.1097/mco.0000000000000847. PubMed PMID: 35788094.

35. Ridgers ND, McNarry MA, Mackintosh KA. Feasibility and Effectiveness of Using Wearable Activity Trackers in Youth: A Systematic Review. JMIR mHealth and uHealth. 2016;4(4):e129. Epub 2016/11/25. doi: 10.2196/mhealth.6540. PubMed PMID: 27881359; PubMed Central PMCID: PMCPMC5143467.

36. Creaser AV, Clemes SA, Costa S, Hall J, Ridgers ND, Barber SE, et al. The Acceptability, Feasibility, and Effectiveness of Wearable Activity Trackers for Increasing Physical Activity in Children and Adolescents: A Systematic Review. International journal of environmental research and public health. 2021;18(12). Epub 2021/07/03. doi: 10.3390/ijerph18126211. PubMed PMID: 34201248; PubMed Central PMCID: PMCPMC8228417.

37. Ridgers ND, Timperio A, Brown H, Ball K, Macfarlane S, Lai SK, et al. Wearable Activity Tracker Use Among Australian Adolescents: Usability and Acceptability Study. JMIR mHealth and uHealth. 2018;6(4):e86. Epub 2018/04/13. doi: 10.2196/mhealth.9199. PubMed PMID: 29643054; PubMed Central PMCID: PMCPMC5917084.

38. Kellett S, Simmonds-Buckley M, Bliss P, Waller G. Effectiveness of Group Behavioural Activation for Depression: A Pilot Study. Behavioural and cognitive psychotherapy. 2017;45(4):401-18. Epub 2017/03/14. doi: 10.1017/s1352465816000540. PubMed PMID: 28287065.

39. Lejuez CW, Hopko DR, Acierno R, Daughters SB, Pagoto SL. Ten year revision of the brief behavioral activation treatment for depression: revised treatment manual. Behavior modification. 2011;35(2):111-61. Epub 2011/02/18. doi: 10.1177/0145445510390929. PubMed PMID: 21324944.

40. Montgomery SA, Asberg M. A new depression scale designed to be sensitive to change. The British journal of psychiatry : the journal of mental science. 1979;134:382-9. Epub 1979/04/01. doi: 10.1192/bjp.134.4.382. PubMed PMID: 444788.

41. Reliability and validity of the Chinese version of Beck Depression Inventory-II among depression patients [Internet]. China: Chinese Mental Health; 2011

42. Shudo Y, Yamamoto T. Assessing the relationship between quality of life and behavioral activation using the Japanese Behavioral Activation for Depression Scale-Short Form. PloS one. 2017;12(9):e0185221. Epub 2017/09/29. doi: 10.1371/journal.pone.0185221. PubMed PMID: 28957346; PubMed Central PMCID: PMCPMC5619729.

43. Yuhui W, Wan L, Jiahu H, Fangbiao T, Maternal DO, Childamp, et al. Development and evaluation on reliability and validity of Adolescent Non-suicidal Self-injury Assessment Questionnaire. 2018.

44. Morin CM, editor Insomnia: Psychological Assessment and Management1993.

45. Craig CL, Marshall AL, Sjöström M, Bauman AE, Booth ML, Ainsworth BE, et al. International physical activity questionnaire: 12-country reliability and validity. Medicine and science in sports and exercise. 2003;35(8):1381-95. Epub 2003/08/06. doi: 10.1249/01.Mss.0000078924.61453.Fb. PubMed PMID: 12900694.

46. Szuhany KL, Otto MW. Efficacy evaluation of exercise as an augmentation strategy to brief behavioral activation treatment for depression: a randomized pilot trial. Cognitive behaviour therapy. 2020;49(3):228-41. Epub 2019/07/31. doi: 10.1080/16506073.2019.1641145. PubMed PMID: 31357916; PubMed Central PMCID: PMCPMC6989384.
