# Supplementary material for: The effect of behavioral activation play therapy in adolescents with depression: A study protocol for a randomized controlled trial
Source: PLoS One. 2024 Jun 20;19(6):e0304084. doi: 10.1371/journal.pone.0304084 (PMC11189190; doi:10.1371/journal.pone.0304084)
Supplement: S3 File — The letter of notification of the review opinion of the Regional Committee for Medical and Health Research Ethics. (PDF) [file pone.0304084.s003.pdf]

伦理审查意见通知函

通知函号：（2023）第（036）号

审查日期	2023.03.27	审查会议地点	广州医科大学附属脑科医院计算机中心培训室
研究项目名称	青少年抑郁症行为激活游戏治疗对青少年抑郁症的干预效果研究		
申办者	/		
研究单位	广州医科大学附属脑科医院	主要研究者	黄小龙
审查文件	1. 研究方案 版本：1.0，日期：2023年3月17日<br>2. 知情同意书 版本：1.0，日期：2023年3月17日<br>3. 病例报告表 版本：1.0，日期：2023年3月17日<br>4. 主要研究者简历<br>5. 风险预案<br>6. 附件（小米手环7pro说明书）		
审查类别	■初始审查 □复审	审查方式	■会议审查 □快速审查
跟踪审查频率	/		
审查委员	本次会议13位委员参会，12位委员参与该项目审查及投票，1位委员回避		
审查意见	经本伦理委员会审查，审查意见为：**作必要的修正后重审**。具体意见如下：<br>1. 方案和知情同意书中补充激活游戏的具体内容。<br>2. 补充可操作的评估自杀意念的方法，甄别出有自杀意念受试者。<br>3. 补充在干预阶段受试者如果出现自杀风险的处置预案。<br>主任委员/被授权者签字：[signature]<br>广州医科大学附属脑科医院伦理委员会（盖章）<br>日期：2023.4.3		
备注	1. 按审查意见修改后的文件，或对审查意见不同观点的陈诉，请提交“复审申请”，方案/知情同意书请注明新的版本号和版本日期，并以阴影和（或）下划线方式标注修改部分，报伦理委员会审查，经批准后执行。<br>2. 不同意/暂停或终止项目，2周内可向伦理委员会就有关事项做出解释或提出申诉。		
声明	本伦理委员会的职责、人员组成、操作程序及记录遵循ICH-GCP、中国的相关法律法规。		

伦理审查批件

批件号：（2023）第（027）号

审查日期	2023.04.24	审查会议地点	广州医科大学附属脑科医院计算机中心培训室
研究项目名称	青少年抑郁症行为激活游戏治疗对青少年抑郁症的干预效果研究		
申办者	/		
临床研究单位	广州医科大学附属脑科医院	主要研究者	黄小龙
审查文件	1. 复审申请<br>2. 研究方案 版本：2.0，日期：2023-4-10<br>3. 知情同意书 版本：2.0，日期：2023-4-10<br>4. 病例报告 版本：2.0，日期：2023-4-10<br>5. 风险预案 版本：2.0，日期：2023-4-10		
审查类别	□初始审查 ■复审	审查方式	■会议审查 □快速审查
跟踪审查频率	/		
审查委员	本次会议 13 位委员参会，12 位委员参与该项目审查及投票，1 位委员回避		
审查意见	经本伦理委员会审查，同意按照复审通过的研究方案、知情同意书等文件进行该项临床研究。本批件有效期为一年。<br><br>主任委员/被授权者签字：[signature]<br>广州医科大学附属脑科医院伦理委员会（盖章）<br>日期：2023.4.27		

声明：本伦理委员会的职责、人员组成、操作程序及记录遵循 ICH-GCP、中国的相关法律法规。

注意：（请仔细阅读）

1. 已批准项目须遵循本伦理委员会批准的方案执行，须符合 GCP 和《赫尔辛基宣言》的原则。
2. 暂停/提前终止临床研究，请及时通知伦理委员会。
3. 发生严重不良事件及影响研究风险受益比的非预期事件，须及时报告本伦理委员会。
4. 对已批准的临床研究方案、知情同意书等材料的任何修改及主要研究者更换等，须及时通知本伦理委员重新审查，获得批准后执行。
5. 发现违反方案情况须及时报告。
6. 根据伦理委员会对跟踪审查频率的意见，无论试验开始与否，请在跟踪审查日到期前 1 个月提出跟踪审查的申请。
7. 完成临床研究，须提交结题报告供伦理委员会审查。
