# Supplementary material for: The effect of behavioral activation play therapy in adolescents with depression: A study protocol for a randomized controlled trial
Source: PLoS One. 2024 Jun 20;19(6):e0304084. doi: 10.1371/journal.pone.0304084 (PMC11189190; doi:10.1371/journal.pone.0304084)
Supplement: S4 File — Translated version of the letter of notification of the review opinion of the Regional Committee for Medical and Health Research Ethics. (PDF) [file pone.0304084.s004.pdf]

TNE AFFLUATED BRAIN HOSPITAL OF GUANGZHOU MEDHCAL UIVEA SITY

IRB, The Ethics Committee of the Affiliated Brain Hospital of Guangzhou Medical

Notification of ethical review

Notification Letter No.:(2023) No. (036)

Review date	2023.03.27	Review meeting location	Training Room of Computer Center, Affiliated Brain Hospital of Guangzhou Medical University
Name of the research project	The effect of behavioral activation play therapy in adolescents with depression: a study protocol for a randomized controlled trial		
The applicant	/		
Research unit	The Affiliated Brain Hospital of Guangzhou Medical University	Principal investigator	Xiaolong Huang
audit-review file	1 . Study protocolVersion: 1.0, dated 17 March 2023<br>2. Informed Consent Form Version: 1.0, dated March 17,2023 3. Case Report Form Version: 1.0, dated 17 March 2023<br>4. Resume of the principal investigator<br>5 . Risk preplan<br>6. Attachment (Mi Band 7 Pro manual)		
Review category	■ Initial review □ review	Review method	■ Conference review of the<br>□ rapid review
Track the frequency of review	/		
juror	At this meeting, 13 members attended, 12 members participated in the project review and voted, and 1 member avoided		
Review opinions	After the review of the ethics committee, the review opinion is: make the necessary amendment and retrial. The specific opinions are as follows:<br>1. The specific content of the activated game is added inthe plan and the informed consent form.<br>2. Supplement actionable methods for assessing suicidal ideation to identify subjects with suicidal ideation.<br>3.Supplement the disposal plan for the risk of suicide during the intervention stage.<br><br>Signature of the Chairman / Authorized Person:		

	Ethics Committee of the Affiliated Brain Hospital of Guangzhou Medical University (seal)<br>州医科大学附属脑科医院 伦理委员会<br>date: 2023. 4. 3
remarks	1. For the revised documents according to the review opinions, or thecomplaints of different opinions on the review opinions, please submit the "review application". The protocol / informed consent form please indicate the new version number and version date, and mark the modified part with shadow and (or) underline, and report to the Ethics Committee for review, and execute after approval.<br>2. If you disagree / suspend or terminate the project you may explain or appeal to the ethics committee within 2 weeks.
statement	The responsibilities, personnel composition, operating procedures and records of the EC shall comply with the relevant laws and regulations of ICH-GCP and China.

Notification of ethical review

Approval Letter No.:(2023) No. (027)

Review date	2023.04.24	Review meeting location	Training Room of Computer Center, Affiliated Brain Hospital of Guangzhou Medical University
Name of the research project	The effect of behavioral activation play therapy in adolescents with depression: a study protocol for a randomized controlled trial		
The applicant	/		
clinical research unit	The Affiliated Brain Hospital of Guangzhou Medical University	Principal investigator	Xiaolong Huang
audit-review file	1. Application for review<br>2. Study protocol<br>3. Informed consent form<br>4. case-reporting<br>5. Risk preplan	Version: 2.0, dated: 2023-4-10<br>Version: 2.0, dated: 2023-4-10<br>Version: 2.0, dated: 2023-4-10<br>Version: 2.0, dated: 2023-4-10	
Review category	Oral initial review ■ review	Review method	■ Conference review of the ☐ rapid review
Track the frequency of review	/		
juror	At this meeting, 13 members attended the meeting, 12 members participated in the review and voting of the project, and 1 member avoided		
Review opinions	After the review, the ethics committee agreed to conduct the clinical study in accordance with the approved study protocol, informed consent and other documents. This approval letter is valid for one year.<br><br>Signature of the Chairman / Authorized Person:		

Ethics Committee of the Affiliated Brain Hospital of Guangzhou Medical University (seal)

广州医科大学附属脑科医院伦理委员会

date: 2023.4.27

Statement: The responsibilities, personnel composition, operating procedures and records of the EC comply with the relevant laws and regulations of ICH-GCP and China.

Note: (please read it carefully)

1. The approved projects shall follow the protocol approved by the Ethics Committee and shall comply with the principles of the GCP and the Declaration of Helsinki.2. To suspend / prematurely terminate the clinical study, please inform the Ethics Committee promptly.

3. The occurrence of serious adverse events and unexpected events affecting the risk-benefit ratio of the study shall be reported to the EC in time.

4. Any modification of the approved clinical study protocol, informed consent form and other materials and the replacement of the principal investigator shall be timely notified to the ethics committee after the approval.

5. Any violation of the scheme shall be reported in time.

6. According to the opinions of the Ethics Committee on the frequency of follow-up review, whether the trial starts or not, please submit the application for follow-up review 1 month before the expiration of the follow-up review date. 7. To complete the clinical study, the concluding report shall be submitted for the ethics committee review.

Address: No.36 Mingxin Road, Liwan District, Guangzhou postcode: 510370 Tel: 020-81268229

Add:36#,Mingxin Road,Fangcun,Liwan District,Guangzhou Pos t Code:510370 Tel:86-020-81268229
