# Supplementary material for: The effect of behavioral activation play therapy in adolescents with depression: A study protocol for a randomized controlled trial
Source: PLoS One. 2024 Jun 20;19(6):e0304084. doi: 10.1371/journal.pone.0304084 (PMC11189190; doi:10.1371/journal.pone.0304084)
Supplement: Attachment — Submitted filename: Response to Reviewers.docx [file pone.0304084.s006.docx]

Dear Editor,

Dear Reviewers,

Paper EMID: 3a7d0f79757a3aad

Paper Title: The effect of behavioral activation play therapy in adolescents with depression: a study protocol for a randomized controlled trial

We would to thank the editor and the two reviewers again for their valuable comments. We have submitted 3 files, including a revised manuscript (labeled 'Manuscript'), a track changes file (labeled 'Revised Manuscript with Track Changes'), and this file, i.e., the response letter (a complete response to the editor and reviewers). In the following, we respond to each of your concerns and recommendations. The line numbers we provided in this response letter refer to the revised manuscript (file labeled 'Manuscript').

To comprehensively respond to all reviewer feedback, we have pasted their comments into this document. Our responses are noted with "Author Response (AR) " and we note the location (lines) of any changes made to the manuscript.

Once again, we sincerely thank you very much for the time and attention you devoted to this manuscript.

Sincerely,

Authors

Response to Reviewers

(EC: Editor’s Comment, RC: Reviewer’s Comment, AR: Author's Response)

Response to the Editor:

EC0: 1. Please ensure that your manuscript meets PLOS ONE's style requirements, including those for file naming. The PLOS ONE style templates can be found at

<https://journals.plos.org/plosone/s/file?id=wjVg/PLOSOne_formatting_sample_main_body.pdf> and

<https://journals.plos.org/plosone/s/file?id=ba62/PLOSOne_formatting_sample_title_authors_affiliations.pdf>

AR0: Thank you for your guidance. We appreciate the links you provided to the PLOS ONE style templates, and had carefully reviewed them to make the necessary adjustments. We ensured that our manuscript aligns with PLOS ONE's style requirements, including those for file naming.

EC1: 2. Thank you for stating the following financial disclosure:

"This study was supported by Science and Technology Program of Guangzhou (grant No. 205171098044), Guangzhou Municipal Key Discipline in Medicine (2021-2023), Guangzhou High-level Clinical Key Specialty, and Guangzhou Research-oriented Hospital."

AR1: We had stated in funding: The funders had no role in study design, data collection and analysis, decision to publish, or preparation of the manuscript (please see details in attachment “Funding”).

EC2: 3. Please provide a complete Data Availability Statement in the submission form, ensuring you include all necessary access information or a reason for why you are unable to make your data freely accessible. If your research concerns only data provided within your submission, please write "All data are in the manuscript and/or supporting information files" as your Data Availability Statement.

AR2: Thank you for your suggestion, according to your suggestion, we added the information you suggested "All data are in the manuscript and/or supporting information files. Deidentified research data will be publicly available on the Clinical Trial Management Public Platform (URL: http://www.medresman.org.cn/) upon completion and publication of the study". The added information is as follows (please see details in lines 614-617 of the manuscript).

EC3: 4. Your ethics statement should only appear in the Methods section of your manuscript. If your ethics statement is written in any section besides the Methods, please delete it from any other section.

AR3: Thank you for your suggestion. We added the "Ethics statement" in the "Methods and design" (please see details in lines 209-213 of the manuscript). Any duplication of the ethics statement in other sections had removed as per your suggestion.

EC4: 5. We note that the original protocol file you uploaded contains a confidentiality notice indicating that the protocol may not be shared publicly or be published. Please note, however, that the PLOS Editorial Policy requires that the original protocol be published alongside your manuscript in the event of acceptance. Please note that should your paper be accepted, all content including the protocol will be published under the Creative Commons Attribution (CC BY) 4.0 license, which means that it will be freely available online, and any third party is permitted to access, download, copy, distribute, and use these materials in any way, even commercially, with proper attribution.

AR4: Thank you for bringing this to our attention. We hereby confirm that the original protocol for “The effect of behavioral activation play therapy in adolescents with depression: a study protocol for a randomized controlled trial” has obtained permission from The Affiliated Brain Hospital of Guangzhou Medical University (please see details in attachment “Declaration”), for publication in your journal. We understand that, according to the PLOS Editorial Policy, the original protocol will be publicly disclosed alongside our manuscript upon acceptance.

EC5: 6. Please review your reference list to ensure that it is complete and correct. If you have cited papers that have been retracted, please include the rationale for doing so in the manuscript text, or remove these references and replace them with relevant current references. Any changes to the reference list should be mentioned in the rebuttal letter that accompanies your revised manuscript. If you need to cite a retracted article, indicate the article’s retracted status in the References list and also include a citation and full reference for the retraction notice.

AR5: Thank you for your suggestion. We checked the references list to ensure that it is complete and correct.

Response to Reviewers:

Reviewer #1

• What are the main claims of the paper and how significant are they for the discipline?

RC1.1: 1. Randomized Controlled Trial (RCT) Design: The RCT is a strong study design for determining the efficacy of interventions. However, the success of an RCT largely depends on its execution, including randomization methods, blinding, and control of confounding factors.

AR1.1: Thank you for your careful evaluation of this manuscript. The requirements of a randomized controlled trial will be strictly followed, including the screening of participants based on the inclusion and exclusion criteria (see details in lines 199-201 of the manuscript), and the enforcement of randomized allocation using the random number table method (see details in lines 271-279 of the manuscript). To ensure assessor-blind, 5 assessors from outside the unit will be recruited to measure the outcomes (see details in lines 284-293 of the manuscript). The effects of potential confounders will be controlled for using analysis of covariance (see details in lines 474-481 of the manuscript).

RC1.2: 2. Single-blind Approach: The study used a single-blind approach, in which outcome assessors were unaware of the group assignment. Although this helps reduce bias, it is not as robust as a double-blind design. In interventions involving physical activity, double-blinding is challenging, but a single-blind design may still introduce bias.

AR1.2: This study will adopt an assessment-blind approach, as blinding the participants and interventionists to the study conditions is not feasible within this behavioral intervention. Therefore, to minimize the impact of information bias, five evaluators responsible for measuring study outcomes will be recruited from external sources and kept unaware of the group assignments. To ensure the blinding of the evaluators to the group assignments, they will not conduct follow-up measurements for the same group (see details in lines 284-293 of the manuscript). We added Fig 3 to present the evaluation arrangements in detail.

Fig 3. Evaluation arrangements

*A, B, C, D, E represent 5 independent evaluators.

RC1.3: 3. Sample Size and Population: A sample size of 200 adolescents appears adequate, but it is important to consider whether this sample is representative of the broader adolescent population, which is not clearly stated in the article.

AR1.3: This study investigates the clinical intervention of a novel therapeutic technique called BAPT. The primary goal of healthcare professionals is to meet the needs of hospital patients. Therefore, we are currently only recruiting participants from the hospital. We may consider expanding the scope of recruitment to the community, schools, etc. in the future. We have provided additional information on this limitation (see details in lines 560-563, 569-570 of the manuscript). In addition, following the recommendation of Reviewer #2, the study has been structured as a randomized controlled trial with three arms: the BAPT group, the BA group, and the GPA group. Additionally, the sample size has been recalculated, resulting in a total of 258 participants (see details in lines 462-467 of the manuscript).

RC1.4: 4. Intervention Complexity: The integration of BA and GPA could add complexity to the intervention, which might impact its replicability in different settings or practicality in routine clinical practice.

AR1.4: Thank you for your valuable feedback. We acknowledge the concern regarding the potential increase in intervention complexity due to the integration of BA and GPA. Initially, we applied BAPT to adolescents with depression in an inpatient setting and found it is feasible and accessible to both therapists and participants. Due to its simplicity and ease of use, trained health workers can use BA. Some scholars had combined BA with PA or improvisation to achieve good results in alleviating depressive symptoms. This confirms that BA can be integrated with other therapeutic techniques (see details in lines 115-116, 122-129, 533-535 of the manuscript and references 19-21, 28-30). Furthermore, the manuscript has been refined to include detailed intervention manuals for BAPT, BA, and GPA, ensuring fidelity and replicability. Therapists will undergo a rigorous two-week training program, which will be assessed using the Behavioral Therapist Competency/Adherence Questionnaires (BTCAQ). Upon completing the program, therapists will receive certification before implementing the interventions. Expert mentors will provide ongoing support through weekly online clinical supervision sessions. To assess fidelity and feasibility, researchers will use the BTCAQ to evaluate random sessions (see details in lines 248-269 of the manuscript). In the future, we will validate, refine, and promote BAPT in various settings.

Potential Limitations and Areas for Improvement

RC1.5: 1. Follow-Up Duration: The long-term effects of BAPT are not clear from the abstract. A longer follow-up period would be beneficial for assessing the sustainability of treatment effects.

AR1.5: Thank you for your suggestion. We have extended the follow-up period to 28 weeks to assess the sustainability of the treatment effects (see details in lines 417 of the manuscript and S1 Fig 1).

RC1.6: 2. Comparison with Existing Treatments: The study compared BAPT with BA only, and not with other standard treatments such as CBT or medication. These comparisons provide a clearer picture of BAPT's relative efficacy of BAPT.

AR1.6: Thank you for your suggestion. As BAPT is a new technique that combines BA with GPA, we would like to confirm our research hypothesis by first experimentally demonstrating that using BAPT is more effective for adolescents with depression than BA or GPA alone. We appreciate your suggestion regarding comparing BAPT with other standard treatments such as CBT or medication. We added a note in the "Advantages and limitations" section of the manuscript (see details in lines 555-558 of the manuscript). While we acknowledge the importance of such comparisons in assessing the relative efficacy of BAPT, our study design focused specifically on evaluating the effectiveness of BAPT compared to BA or GPA in the context of clinical intervention. However, we agree that future research could explore comparative effectiveness studies involving a broader range of treatment modalities such as CBT or medication to provide a more comprehensive understanding of BAPT's relative efficacy (see details in lines 567-572 of the manuscript). We will consider incorporating such comparisons in future investigations to enhance the depth of our findings.

RC1.7: 3. Measurement of Outcomes: The Reliance on self-reported measures for some outcomes could introduce bias. Objective measures, where feasible, would strengthen this study's findings.

AR1.7: Thank you for your reminder. We measured the primary outcome of depressive symptoms by using the MADRS (Objective measures) (see details in lines 355-363 of the manuscript).and the BDI-II-C (self-reported measures). Additionally, we monitored participants' physical activities (including volume and intensity, etc.) (see details in lines 436-440 of the manuscript). In subsequent studies, we will aim to incorporate more objective measurements.

RC1.8: 4. Consideration of Confounding Variables: Adolescent depression can be influenced by numerous social, environmental, and genetic factors. This study accounted for these potential confounders in the analysis.

AR1.8: Thank you for bringing up this important consideration. We are committed to addressing potential confounding variables in our analysis. To achieve this, one-way ANOVA will be used to identify variables (e.g., socio-demographic information, etc.) that have statistically significant differences (p < 0.05) among the three groups of subjects at baseline. These potential confounders will then be included as covariates in the ANOVA model for analysis (see details in lines 474-481 of the manuscript).

Implications and Significance

RC1.9: 1. Innovative Approach: This study's focus on integrating gamified physical activities into behavioural activation therapy is innovative and could lead to more engaging treatment modalities for adolescents.

AR1.9: Thank you for acknowledging the innovative approach of our study, which focuses on integrating gamified physical activities into behavioural activation therapy for adolescents. This approach aligns with the psychological development of adolescents (see details in references 31) and the WHO's guidelines on physical activity for this population (see details in references 14). We agree that this integration has the potential to create more engaging treatment modalities for this population. We appreciate your recognition of the novelty of our approach and its potential implications for adolescent mental health treatment.

RC1.10: 2. Potential Clinical Impact: If BAPT is effective, it could offer a valuable tool for treating adolescent depression, potentially improving adherence and outcomes.

AR1.10: Thank you for highlighting the potential clinical impact of our study. We fully recognize the significance of BAPT as a potential valuable tool for treating adolescent depression. We agree that if BAPT proves to be effective, it could indeed improve adherence to treatment and lead to better outcomes for adolescents struggling with depression. Therefore, we included the Custom Acceptance and Compliance Questionnaire (CACQ) to assess acceptance and compliance, which consists of seven items scored on a five-point Likert scale. The questionnaire assesses participants' satisfaction, willingness to continue receiving services, and willingness to recommend services. Adherence will also be analyzed (see details in lines 395-399 of the manuscript). We appreciate your recognition of the importance of our research in this context.

RC1.11: 3. Contribution to the Field: This research could contribute significantly to the understanding of non-pharmacological interventions in adolescent mental health, an area that requires more empirical data.

AR1.11: Thank you for recognizing the potential contribution of our research to the field of non-pharmacological interventions in adolescent mental health. We agree that there is a need for more empirical data in this area, including representativeness of sample, meticulous control of confounding variables, reproducibility of trial, etc. We are committed to contributing to the understanding of effective interventions for adolescent mental health. We appreciate your acknowledgment of the significance of our research in addressing this important gap in the literature.

RC1.12: While this study presents an innovative approach that has the potential to make significant contributions to the field of adolescent mental health, it is essential to carefully consider its design, execution, and potential limitations. These results should be interpreted within the context of these factors to understand the true efficacy and applicability of BAPT.
AR1.12: Thank you for your insightful evaluation of our study and for recognizing the innovative approach we have taken in integrating gamified physical activities into behavioral activation therapy (BAPT) for treating adolescent depression. We take your comments regarding the design, execution, and potential limitations of our study seriously. We have endeavored to address these factors by implementing rigorous methodology, including the improvement of study design, methods of recruitment, therapist eligibility, intervention fidelity, blinding (see details in “Method and design” of the manuscript), control for confounding variables (see details in lines 474-481 of the manuscript) and ensuring the use of both objective and self-reported outcome measures. Moving forward, we remain committed to refining our approach and addressing any identified limitations to ensure the validity and applicability of our findings once again (see details in lines 567-570 of the manuscript), thank you for your valuable feedback, which will undoubtedly contribute to the continued improvement and impact of our research in the field of adolescent mental health intervention.

• Are the claims properly placed in the context of the previous literature? Have the authors treated the literature fairly?

RC1.13: Comparative Analysis: This study could be strengthened by explicitly comparing the proposed BAPT method with existing treatments in terms of efficacy, side effects, and adherence rates.

**AR1.13:** Thank you very much for your suggestions. We have made revisions to the manuscript accordingly. We added two outcomes, namely the Short Inventory of the Assessment of Negative Effects (SIAN) and The Negative Effects Questionnaire (NEQ), to assess the side effects post-intervention (see details in lines 405-416 of the manuscript). Additionally, an analysis of adherence rates was incorporated into the Custom Acceptance and Compliance Questionnaire (CACQ) (see details in lines 395-399 of the manuscript). The assessment of efficacy continues to utilize several outcomes originally described in the manuscript.

RC1.14: Broader Literature Scope: Depending on the full content of the paper, it would be beneficial to include a wider range of studies, particularly those with opposing viewpoints or results, to provide a more comprehensive view of the current state of research.
AR1.14: Thank you for the valuable suggestions. In the revised manuscript, we have explicitly compared the BAPT method with other psychotherapies in terms of efficacy, economic cost, and adherence (see details in lines 32-34, 88-91, 111-113, and 508-532 of the manuscript). Additionally, we have expanded the scope of literature to critically discuss the current efficacy of BA, PA and the combination of BA with PA to ensure a comprehensive and fair review of the research landscape (see details in lines 113-115, 130-134, 496-507 and 527-531 of the manuscript).

RC1.15: Longitudinal Studies and Meta-Analyses: The inclusion and discussion of longitudinal studies or meta-analyses in the field would provide deeper insights into the long-term effectiveness of treatments for adolescent depression.
AR1.15: Thank you for your valuable suggestion. To delve deeper into the long-term efficacy of treatments for adolescent depression, we have incorporated several longitudinal studies and meta-analyses into our analysis (see details in lines 508-532 of the manuscript), as evidenced by the references cited. These additions provide critical insights into the trajectory and determinants of treatment effects over extended periods. We are confident that the inclusion of these supplementary materials enhances the comprehensiveness of our study and fosters a more nuanced understanding of the enduring impact of interventions for adolescent depression.

• Do the data and analyses fully support the claims? If not, what other evidence is required?

RC1.16: No data sets have been described

AR1.16: We apologize for the lack of description regarding the data sets in the manuscript. At this point, no data sets have been generated. However, we are committed to conducting meticulous analyses upon completion of the data collection phase. Deidentified research data will be publicly available on the Clinical Trial Management Public Platform (URL: http://www.medresman.org.cn/) upon completion and publication of the study.

• PLOS ONE encourages authors to publish detailed protocols and algorithms as supporting information online. Do any particular methods used in the manuscript warrant such treatment? If a protocol is already provided, for example for a randomized controlled trial, are there any important deviations from it? If so, have the authors explained adequately why the deviations occurred?

RC1.17: The authors must provide a detailed guide on how recruitment will be performed and explain how the fidelity, feasibility, and acceptability of the intervention will be achieved.

AR1.17: Thanks for your suggestions. We acknowledge the significance of meticulously addressing recruitment, treatment fidelity, feasibility, and acceptability. Accordingly, we have adjusted in the following areas:

Recruitment: We have adjusted our recruitment methods to provide a more detailed description (see details in lines 185-195 of the manuscript). Given that BAPT is an innovative approach, we prioritized recruiting patients visiting the clinic. We have explained the limitations of our sample in the study (see details in lines 556-559 of the manuscript).

Treatment Fidelity, feasibility, and acceptability: We recruited non-psychology healthcare professionals as therapists (see details in lines 203-207 of the manuscript) and provided them with treatment manuals. Therapists are required to undergo training and assessment before conducting interventions. Additionally, we will utilize the BTCAQ to evaluate the fidelity, adherence, feasibility, and acceptability of the intervention plan (see details in lines 248-269 of the manuscript).

• If the paper is considered unsuitable for publication in its present form, does the study itself show sufficient potential that the authors should be encouraged to resubmit a revised version?

RC1.18: Upon addressing any outstanding queries and incorporating the responses, this paper will be deemed suitable for publication.

AR1.18: We are grateful for the opportunity to improve our work and will resubmit a revised version of the manuscript in line with your recommendations.

• Are original data deposited in appropriate repositories and accession/version numbers provided for genes, proteins, mutants, diseases, etc.?

RC1.19: None seen

AR1.19: Thank you for your inquiry regarding the deposition of original data. We appreciate your attention to this matter. In our manuscript, we have outlined our data management plan, which involves the utilization of Case Report Forms (CRF) to document participant information and symptoms. These data will be securely stored on the Clinical Trial Management Public Platform (URL: http://www.medresman.org.cn/), with stringent measures in place to ensure data integrity and privacy protection upon completion and publication of the study. Access to the data will be restricted to relevant members of the research group, and identifying information will be removed during the data entry process to safeguard participant anonymity (see details in lines 448-457of the manuscript). Additionally, the original CRF will be securely stored and monitored by a dedicated physician to ensure data completeness and accuracy (see details in lines 457-460 of the manuscript). We believe these measures align with best practices for data management and protection in research settings.

Furthermore, regarding data sharing, we are committed to promoting transparency and reproducibility in research. While our primary data will be securely stored on the Clinical Trial Management Public Platform (URL: http://www.medresman.org.cn/) (see details in lines 614-617 of the manuscript), we recognize the importance of facilitating access to our findings for the broader scientific community. Therefore, upon request, we are willing to provide access to de-identified data sets to qualified researchers, in accordance with applicable data protection regulations and the Creative Commons Attribution (CC BY) 4.0 license. We believe that fostering data sharing will enhance the impact and rigor of our research findings.

• Does the study conform to any relevant guidelines such as CONSORT, MIAME, QUORUM, STROBE, and the Fort Lauderdale agreement?

RC1.20: Yes, it conforms to CONSORT

AR1.20: Thank you for confirming that our study conforms to CONSORT guidelines.

• Are details of the methodology sufficient to allow the experiments to be reproduced?

RC1.21: A more in-depth description of the methods is required.

AR1.21: Thank you for your feedback. We have revised the “Methods and Design” section of the manuscript to provide a more detailed description of the study. This includes information on the recruitment (see details in lines 185-195 of the manuscript), therapist eligibility (see details in lines 203-207 of the manuscript), assent and informed consent (see details in lines 215-226 of the manuscript), risks associated with physical activity (see details in lines 239-247 of the manuscript), intervention fidelity (see details in lines 248-269, 298-300 of the manuscript), blinding (see details in lines 281-293 of the manuscript), intervention (see details in lines 296-300, 321-325 of the manuscript), efficacy evaluations (see details in lines 395-416 of the manuscript), data management (see details in lines 448-451, 457-458 of the manuscript), sample size calculation (see details in lines 460-465 of the manuscript), data analysis (see details in lines 470-485 of the manuscript), demographic and clinical data (see details in lines 428-429 of the manuscript).

• Is the manuscript well organized and written clearly enough to be accessible to non-specialists?

RC1.22: With minor edits to the language used, it will be suitable.

AR1.22: We appreciate your assessment that with minor language edits, the manuscript will be suitable for non-specialists. We have carefully reviewed your suggestions and made the necessary revisions to improve the manuscript's accessibility. These revisions include simplifying the language and optimizing the paragraph structure. We believe these changes enhance the manuscript's readability and make it more accessible to a broader audience.

• Is it your opinion that this manuscript contains an NIH-defined experiment of Dual Use concern?

RC1.23: It is highly unlikely that this manuscript contains an NIH-defined experiment on dual-use research of concern.

AR1.23: Thank you for your assessment. We agree that it is highly unlikely that our manuscript contains an NIH-defined experiment on dual-use research of concern.

Reviewer #2

RC2.1: "...On the basis of conventional treatment and care, we will 72 compare the outcomes of BAPT and BA. The intervention group and control group will 73 receive nine sessions of BAPT or BA, respectively"

Comment: Since the effect size of BA is already known to be very small, why not have three treatment arms comparing BAPT, BA, and GPA.

AR2.1: Thank you for your valuable suggestion. Following your recommendation, we have revised the trial to incorporate three treatment groups: the BAPT group, the BA group, and the GPA group (see details in lines 165-166 of the manuscript). Consequently, we updated the corresponding flowchart (see details in annex S1 Fig 1), data analysis (see details in lines 470-485 of the manuscript), and sample size calculation method (see details in lines 463-467 of the manuscript).

RC2.2: You need a dedicated section on ethical considerations in this protocol. Describe in detail how the double vulnerabilities of adolescence and depression will be handled to prevent further harm and how voluntariness will be protected. Also minors cannot give informed consent but rather assent, the protocol only talks about informed consent.

AR2.2: Thank you for your guidance. In accordance with your suggestions, we have incorporated a dedicated section entitled "Ethics statement" into the manuscript, providing detailed elaboration on ethical considerations (see details in lines 209-213 of the manuscript). Furthermore, we have integrated "Assent and informed consent" within this section to accurately denote that the minors are only capable of providing assent (see details in lines 215-226 of the manuscript). Additionally, we have addressed the risks associated with "Self-harm and suicide Ideation", as well as "Risks associated with physical activity", aiming to mitigate further harm to adolescent depression patients (see details in lines 228-247 of the revised manuscript).

RC2.3: WHO defines adolescents as individuals between ages 10-19. Explain your choice of ages 12-17.

AR2.3: Thank you for your comment. While it is true that the WHO defines adolescents as individuals aged 10-19, our selection of the age range 12-17 is grounded in several considerations pertinent to our study's objectives and target population.

Firstly, compared to adolescents aged 10-19, those aged 12-17 exhibit more homogeneous levels of cognitive ability, as well as similar levels of acceptance, understanding and integration of the intervention sessions. Additionally, individuals aged 10-11 are typically still in childhood and may have limited cognitive maturity and participation, while individuals aged 18-19 are legally considered adults according to the Civil Code of the People's Republic of China.

Secondly, constraining the age range to 12-17 facilitates the establishment of a more homogenous study cohort in terms of developmental stage, potentially minimizing confounding variables and enhancing the internal validity of our findings.

Thirdly, the age range of 12-17 corresponds to the widely recognized period of adolescence within various clinical and research domains, particularly evident in psychiatric and psychological literature. This age bracket aligns with significant developmental transitions and challenges experienced during this pivotal phase. Focusing on this narrower age span allows for a more targeted exploration of the effects of BAPT on adolescents during the early to mid-adolescent stages of puberty.

Once again, we appreciate the opportunity to provide explanation.

RC2.4: Specialized psychologists will be used to deliver the intervention. How do you eliminate the chances that there is psychological knowledge/skills transfer during the activities, such that at the end of the activities you can be sure that it is indeed BAPT and not interaction with the psychologists (in a novel, play setting) that is causing any effect recorded.

AR2.4: Thank you for your reminder. We have recruited non-psychological professionals to deliver the treatment (see details in lines 203-207 of the manuscript), who will be homogeneously trained and instructed not to bring any psychological knowledge or skills other than BAPT to the interventions, to ensure the consistency of the interventions. In addition, we have programs in place to monitor the fidelity of the intervention (see details in lines 248-269, 298-300 of the manuscript).

RC2.5: line 261-262: How will you control for the effect of possible psychological intervention received up until the point of hospitalisation and recruitment into the study?

AR2.5: Thank you for your comments. To control for the potential impact of psychological interventions received prior to the study, we have implemented the following measures:

Firstly, at baseline we will assess the type, frequency and duration of psychological interventions received by participants prior to study and take this into account as a control variable (see details in lines 428-429 of the manuscript).

Secondly, we will rigorously implement a randomized grouping procedure to ensure that participants who have received psychological interventions are allocated similar probabilities across the three groups. At the same time, we included an exclusion criterion for potential participants who are currently undergoing other forms of psychotherapy to ensure the homogeneity of the study sample (see point 6 of the Exclusion criteria in lines 199 of the manuscript). In addition, participants will not receive other forms of psychological treatment during the study cycle (see details in lines 298-300 of the manuscript).

Finally, we plan to conduct subgroup analyses and sensitivity analyses to assess the potential impact of the psychological intervention on the study outcomes and to determine the extent of its influence on the results.

We believe that these measures will help reduce potential biases that may arise from the psychological intervention and ensure the reliability and validity of the study results. Thank you again for your guidance and advice.

RC2.6: Line 481-482: I think this is an over generalization of adolescents' wishes and preferences. It is assumed here that the adolescents are a homogenous group. Consider having some qualitative interviews with at least some of the participants concerning their views on physical activity.

AR2.6: Thank you for your reminder. We have revised the manuscript to provide a more accurate description (see details in lines 535-540 of the manuscript). In line with the existing literature, physical activities that include elements of play, rules, adventure, excitement, and competition are consistent with the psychological development of adolescents (see details in reference 31). What's more, GPA involves moderate to high intensity physical activity, in line with WHO recommendations for physical activity in adolescents (see details in reference 14). Through preliminary explorations, we have learned about the acceptability of GPA among adolescents with depression, hence the undertaking of this trial. Following your suggestion, to attain a more precise comprehension of the physical activity preferences among adolescents with depression, we incorporated a qualitative interview at baseline to evaluate their inclinations towards physical activity (see details in lines 399-405 of the manuscript). Your feedback is greatly appreciated, and we are committed to enhancing the richness and depth of our research through incorporating qualitative data.

According to the reviewer’s comments, we have revised the manuscript extensively. If there are any other modifications we could make, we would like very much to modify them and we really appreciate your help. We hope that our manuscript could be considered for publication in your journal. Thank you very much for your help.
